# Detection of *Cryptosporidium* spp. and *Giardia* spp. in Environmental Water Samples: A Journey into the Past and New Perspectives

**DOI:** 10.3390/microorganisms10061175

**Published:** 2022-06-07

**Authors:** Marie-Stéphanie Fradette, Alexander I. Culley, Steve J. Charette

**Affiliations:** 1 Institut de Biologie Intégrative et des Systèmes (IBIS), Université Laval, Québec City, QC G1V 0A6, Canada; alexander.culley@bcm.ulaval.ca (A.I.C.); steve.charette@bcm.ulaval.ca (S.J.C.); 2 Département de Biochimie, de Microbiologie et de Bio-Informatique, Faculté des Sciences et Génie, Université Laval, Québec City, QC G1V 0A6, Canada; 3 Centre de Recherche en Aménagement et Développement du Territoire (CRAD), Université Laval, Québec City, QC G1V 0A6, Canada; 4 Groupe de Recherche en Écologie Buccale (GREB), Faculté de Médecine Dentaire, Université Laval, Québec City, QC G1V 0A6, Canada; 5 Centre de Recherche de l’Institut Universitaire de Cardiologie et de Pneumologie de Québec, Québec City, QC G1V 0A6, Canada

**Keywords:** *Cryptosporidium*, *Giardia*, detection, water samples, U.S. EPA Method 1623.1, molecular biology

## Abstract

Among the major issues linked with producing safe water for consumption is the presence of the parasitic protozoa *Cryptosporidium* spp. and *Giardia* spp. Since they are both responsible for gastrointestinal illnesses that can be waterborne, their monitoring is crucial, especially in water sources feeding treatment plants. Although their discovery was made in the early 1900s and even before, it was only in 1999 that the U.S. Environmental Protection Agency (EPA) published a standardized protocol for the detection of these parasites, modified and named today the U.S. EPA 1623.1 Method. It involves the flow-through filtration of a large volume of the water of interest, the elution of the biological material retained on the filter, the purification of the (oo)cysts, and the detection by immunofluorescence of the target parasites. Since the 1990s, several molecular-biology-based techniques were also developed to detect *Cryptosporidium* and *Giardia* cells from environmental or clinical samples. The application of U.S. EPA 1623.1 as well as numerous biomolecular methods are reviewed in this article, and their advantages and disadvantages are discussed guiding the readers, such as graduate students, researchers, drinking water managers, epidemiologists, and public health specialists, through the ever-expanding number of techniques available in the literature for the detection of *Cryptosporidium* spp. and *Giardia* spp. in water.

## 1. Introduction

The production of drinkable water requires the monitoring of several key parameters, such as the presence of chemical compounds, humic acid concentration, turbidity, and microbial content in raw water. To reduce the presence of waterborne pathogenic agents below a tolerable risk level, a series of treatments are applied to the raw water entering the treatment plant to either eliminate or inactivate them. Although the sequence of procedures used is treatment plant-dependent, a general pattern emerges that can be taken as a guideline [1].

First, the water is roughly screened to get rid of macroscopical debris (ex.: branches, aquatic animals, etc.). Secondly, several chemical products (coagulants and flocculants) are added to modify surface electrophysical charges on the particle and attract them to stick to one another, creating denser flocs. Thirdly, these flocs are left to sink to the bottom of large water pools, where they are collected. The water free of flocs is then filtered on a layered granular medium of progressively smaller pore sizes to collect residual matter. Once out of the filters, the water is then disinfected either chemically (ex.: free-chlorine, combined chlorine, chlorine dioxide, ozone) and/or physically (ex.: ultraviolet rays). Once these steps have been completed, the water is then considered potable and is delivered through pipes to customers [1].

Among the microorganisms under surveillance in water treatment, the most famous is likely *Escherichia coli*, a Gram-negative coccobacillus which is a strong indicator of fecal pollution. In order to monitor the abundance of *E. coli* in water samples, several methods are currently used, such as the filtering-membrane protocol or the commercialized Colilert kit [2]. These methods are standardized to be used in environmental analysis laboratories and are quite user-friendly to apply.

Although basic treatments applied at water treatment plants have proven to be effective against common waterborne pathogens such as bacteria and viruses, they do not work as efficiently against cyst-forming protozoa. Their ability to form cysts grants them an increased resistance to stress and to chlorine-based treatments in particular [3]. In fact, previous experiments have demonstrated that ozone and ultraviolet treatments tend to be the most efficient ways to inactivate them, while filtration on a granular medium eliminates significant quantities of cysts [3,4,5]. The most common examples in this category are *Cryptosporidium* spp. and *Giardia* spp., two parasitic protozoa responsible for gastrointestinal symptoms in humans as well as in several animal genera such as cattle [6,7,8,9,10,11], birds [12,13,14], deer [15,16], rodents [17,18,19,20], cats [21,22], dogs [22], snakes [23,24] and other mammals [25].

Throughout their life cycle, these two protozoa can form cyst conformations (oocyst for *Cryptosporidium* and cyst for *Giardia*) as a way of transmitting themselves from one host to the next. The cyst is ingested by the host either through contaminated water and food or by a fecal-oral transmission [26,27,28,29,30]. Once the cyst meets the specific physicochemical conditions of the small intestine, it ruptures to free infectious particles called sporozoites for *Cryptosporidium* and trophozoites for *Giardia*. *Giardia*’s trophozoites bind to the host epithelial cells to proliferate. During the infection, *Cryptosporidium* binds to the host cells and form a parasitic vacuole with the cell membrane [31]. A *Cryptosporidium* oocyst contains four sporozoites, each of which contains one copy of the genome [32,33]. *Giardia*’s cyst holds two undivided trophozoites that split once the cyst has opened. Each trophozoite possesses two identical nuclei with an amount of genome copies reported between four and twelve [34]. Once inside the host’s cell, *Cryptosporidium* sporozoites reproduce asexually then sexually before producing new oocysts [35]. *Giardia* trophozoites reproduce asexually while still bound to the intestinal cell line and ultimately produces new cysts. When physicochemical conditions change, (oo)cysts are excreted via feces into the environment and stay dormant until ingested by the next host. For both parasites, an average of 10 (oo)cysts ingested is required to provoke disease in a human individual [36,37].

Among the data available, it is documented that between 2011 and 2016, approximately 239 outbreaks were caused by *Cryptosporidium* spp. worldwide. [38]. In the United States, the etiological agent in 30,000 cases per year is attributed to either *Cryptosporidium* spp. or *Giardia* spp. [38]. Their illnesses (called cryptosporidiosis and Giardiasis, respectively) are generally characterized by watery stools, dehydration, nausea, vomiting and abdominal cramps, but they can also be asymptomatic in some individuals [39,40]. Although the diseases caused by these organisms are mostly self-healing, the severity and length of these diseases are known to be influenced by the fitness of the host’s immune system [41,42]. For immunocompromised or vulnerable populations, these infections can become chronic or life-threatening [39,40]. Both microorganisms are capable of zoonotic transmission, depending on the host species and parasites involved [43,44]. Therefore, in environments where wildlife and/or livestock can interact with water sources, events of zoonosis could be a major issue if the water is not sufficiently treated before distribution [45].

The fact that little can be done to reduce the contamination of water sources with *Cryptosporidium* spp. or *Giardia* spp. highlights the importance of taking steps to maximize inactivation during treatment, leading to the production of potable water. However, to apply effective and sufficient treatments, it is essential to have a reliable and extensive knowledge of their abundance, identity, viability, and infectivity in raw water sources. Up to now, several methods have been developed to determine these variables, each with its advantages and disadvantages.

First, this review will present the current standard method for the detection of *Cryptosporidium* spp. and *Giardia* spp. from water sources, known as U.S. EPA Method 1623.1. Secondly, other promising methods developed to detect and/or quantify *Cryptosporidium* spp. and *Giardia* spp. from water samples will be reviewed with a strong emphasis on the most recent molecular techniques. Finally, the pros and cons of each of these approaches (Method 1623.1 and molecular methods) will be discussed and compared.

## 2. U.S. EPA Method 1623.1

### 2.1. What Is the U.S. EPA Method 1623.1?

**Figure 1:**
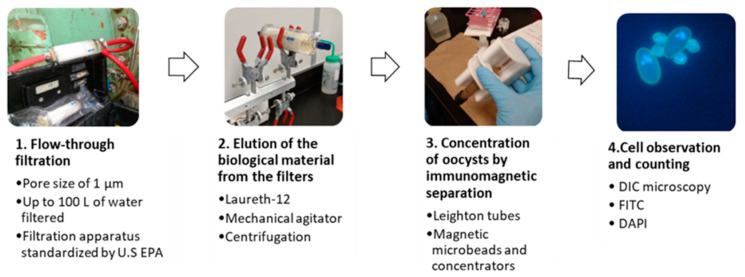
Key steps of the U.S. EPA Method 1623.1. The main steps of the U.S. EPA Method 1623.1 are: (1) the filtration of up to 100 L of the sample with an approved apparatus by the U.S. EPA with a pore size of 1 µm, (2) the elution of the biological material from the filters with mechanical agitation and centrifugation, (3) the concentration of the (oo)cysts by immunomagnetic separation and (4) the observation of the samples by microscopy (DIC followed by fluorescence microscopy with fluorescent molecules DAPI and FITC).

The U.S. EPA Method 1623.1 takes place in four major steps (summarized at Figure 1): the filtration of the water sample, the elution of the biological matter collected on the filter, the concentration of the cysts by immunomagnetic separation (IMS), and the microscopic analysis of the concentrated material. Each of these steps will be described briefly below. For a more detailed description of the protocol, refer to the original U.S. EPA protocol [46].

First, the choice of the sampling site must be made depending on the target of the study (e.g., raw water entering a treatment plant, water exiting a wastewater treatment plant, etc.). But, whatever the sampling site, it must be kept in mind that the following criteria must always be met during sample collection: a continuous flow of 2 L/min, appropriate fittings to plug the filtration apparatus, and a water input of a sufficient volume (from 10 to 50 L).

Once the sampling site has been chosen and the flow rate has been adjusted to 2 L/min, the tubes used for the filtration must be rinsed with a volume of at least 25 L of the sample to clear potential clumps. Then, a U.S. EPA-approved filtration apparatus (ex: an EnviroChek 1 µm pore-size filtration capsule by Pall Corporation, New York, NY, USA) is attached to the conditioned tubes before filtration is initiated. Approximately 50 L is expected to pass through the filter, but care must be taken to monitor the pressure inside the filtration capsule to avoid breakage (must be below 30 psi). Also, it is important to monitor the water flow throughout the period of filtration to keep it as close to the standardized flow rate as possible. Once the filter is clogged, the apparatus can be unplugged, and the capsule is kept cold (2–8 °C) until further processing at the appropriate facility.

The material retained on the filters is eluted by the addition of an elution solution that is dependent on the composition of the filter recommended by the manufacturer (ex: Laureth-12 10% in the case of the EnviroChek 1 µm pore-size filtration cartridge) and agitation of the filtration capsule with a laboratory shaker (Pall Corporation, New York, NY, USA) approved by the U.S. EPA. The elution may also be preceded by a pre-elution step with sodium hexametaphosphate 5% (*w*/*v*) that acts as a chemical dispersant by modifying the surface charge of the filter [47]. Also, a second elution step is suggested to maximize recovery of the biological material from the filter. The eluate is then transferred into a conical tube and centrifuged at 1500× *g* for 15 min. The pellet is recovered, resuspended in 5 mL of ultrapure water (or phosphate-buffered saline with a detergent such as Tween80) per 0.5 mL of pellet and transferred in a Leighton tube for the next step.

Following the elution from the filter, immunomagnetic separation is done using a kit such as the Dynabeads™ GC-Combo (Applied Biosystems, Waltham, MA, USA). Magnetic beads are added to the Leighton tubes. These beads are linked to antibodies recognizing either a *Cryptosporidium* or *Giardia* surface antigen. After a contact time of about an hour, the Leighton tube is placed on a concentrator (such as Dynabeads™ MPC™-1, Applied Biosystems, MA, USA), which is essentially a magnet to separate the metallic beads from the rest of the eluate. The beads attracted to the magnet are transferred into a 1.5 mL microtube, where the dissociation of the beads from the parasites is performed. Briefly, HCl 0.1 N is added to the microtube, which is vortexed and then placed in a second concentrator (such as Dynabeads™ MPC™-S, Applied Biosystems, MA, USA). After a short reaction time (12 min), the magnetic strip of the concentrator is adjusted to recover the beads (freed from the parasites) on the side of the microtube. NaOH 1 N is then added to neutralize the pH of the solution containing the (oo)cysts. The complete volume of liquid at the bottom of the microtube (approximately 75 µL) is transferred onto a glass slide for microscopic observation and the slide is left to dry at 37 °C.

**Figure 2:**
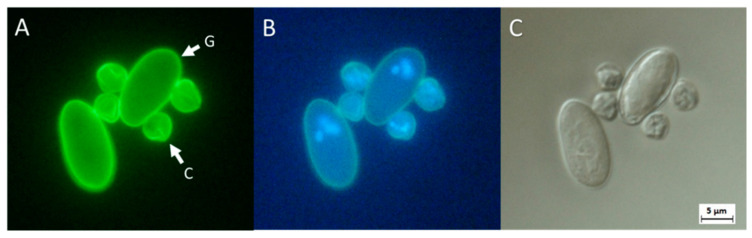
Microscopic observation of *Cryptosporidium* spp. and *Giardia* spp. Cells 4–6 µm in diameter in these pictures belong to the *Cryptosporidium* genus while cells 8–18 µm long by 5–15 µm wide are identified as *Giardia* spp. (**A**) A bright green fluorescence can be seen on the cells’ periphery with the use of two antibodies specific to each parasite linked to FITC. (**B**) The DAPI marker allows the identification of nuclei within the cells. *Cryptosporidium* spp. is known to possess four nuclei in its oocyst configuration. *Giardia* spp. cysts contain two cells that are still linked, each of which has two nuclei. Therefore, two to four nuclei are expected to be observed according to the orientation of the cyst. (**C**) Under differential interference contrast, *Cryptosporidium* spp. oocysts can be seen as spherical structures with rough edges. *Giardia* spp. cysts have an irregular shape most often perceived as ovoid.

As soon as the slides are dried, absolute methanol is pipetted onto each slide. Then, antibodies tagged to fluorescein isothiocyanate (FITC) fluorochrome is added to the slides, left to react for 15 min and the excess solution removed. Fixing buffer (from the Dynabeads kit) is added, left to react for 2 min and the excess solution is also removed. The same thing is done with the 4′,6-diamidino-2-phenylindole (DAPI) fluorochrome solution before adding more of the fixing buffer. Finally, the mounting medium is added, and the slides are sealed with nail polish. Microscopy must be performed on the slides within seven days after their preparation. Slides can be stored in a humid, dark room at 2–8 °C between observations. First, to identify (oo)cysts, a search for FITC fluorochrome-stained for round/ovoid apple-green brilliant structures (5–18 µm in diameter for *Giardia* and 4–6 µm for *Cryptosporidium*) is done at a magnification of 200×. Cells that meet these first criteria are then examined with DAPI fluorochrome at 400× to determine whether nuclei are present (up to four nuclei in both protozoa). It is important to note that the absence of nuclei does not necessarily exclude a positive identification of either parasite. At last, the slides are observed with Differential Interference Contrast at 1000× to determine if the particle examined belongs to either genus of the target protozoa. An example of both protozoa in fluorescence microscopy and in Differential Interference Contrast can be found at Figure 2. As an additional validation of the identification of a cell as a *Giardia* or a *Cryptosporidium* (oo)cyst, a positive control such as ColorSeed™ (BioPoint, Sydney, Australia) can be used for comparison, which are inactivated (oo)cysts stained with red fluorescence (Texas Red). Since the fluorescence of the control (oo)cysts is red, controls and sample cells, which are fluorescent green (FITC) or blue (DAPI), can be easily distinguished.

### 2.2. Pros and Cons of Using U.S. EPA Method 1623.1

A synthesized version of these pros and cons is presented in Table S1.

#### 2.2.1. Pros

Using this standard method for water analyses has several advantages including the possibility of concentrating large volumes of water (up to 100 L). Being able to filter such a large sample volume and then concentrate it with the use of immunomagnetic separation grants a superior collection power. This permits the user to analyze a bigger volume of the water entering the treatment plant, which results in a better assessment of the concentration of *Cryptosporidium* and *Giardia* (oo)cysts. Moreover, one of the strong points of the U.S. EPA Method is its detection limit of one oocyst per 100 L. According to the World Health Organization (WHO), in order to stay below the risk assessment level of 10^−6^ disability-adjusted life years for these protozoa, raw water should contain approximately 10–30 (oo)cysts per 100 L and achieve at least a 3-log reduction at the water treatment plant [48,49]. Therefore, being able to detect such a low number of (oo)cysts from water samples with the U.S. EPA 1623.1 allows compliance with this guideline. The use of microscopy for quantification avoids the biases associated with PCR amplification. Also, the use of several fluorescent dyes simultaneously gives more confidence in the identification. With this approach, the examiner can thus evaluate whether an object is an (oo)cyst and quantify the number of positive cells on each slide.

#### 2.2.2. Cons

Several aspects of the U.S. EPA Method 1623.1 make it a non-optimal technique. The first disadvantage, especially when considering the monitoring of water quality of municipalities with limited resources, is its high cost (approximately 1000$ per sample). Many apparatuses and reagents are required to perform the U.S. EPA protocol correctly. It also requires a significant amount of time. Therefore, several towns and cities may choose not to use this method but rather rely on the concentration of indicator microorganisms (ex: coliform bacteria) to infer the presence of these parasites. This leads to incomplete monitoring of these protozoa across large territories and could ultimately result in many people being vulnerable to infection.

The second well-documented disadvantage of Method 1623.1 is the low recovery of (oo)cysts [50]. It has been shown that the recovery rate can be as low as 50% [51], which makes it unreliable. It was documented that from 8 to 14% of parasites were lost following the centrifugation step of this protocol when compared with samples of the same composition submitted only to IMS and fluorescence microscopy [52]. The same study also found a loss of 29 to 34% of parasites attributable to the filtration and elution protocol by comparison with identical samples but submitted to centrifugation, IMS and fluorescence microscopy. It is also important to underline that although the immunomagnetic separation protocol is useful in removing a large proportion of the non-target biological material, this process can result in cross-reaction, especially with other eukaryotic organisms such as algae, yeast, ciliates and with cellular debris [53,54]. Cross-reaction can also happen during staining with fluorescent dyes such as FITC [53]. Therefore, since many of these organisms have a morphology similar to that of *Cryptosporidium* and *Giardia* cysts, the identification by microscopy can be more difficult even with IMS and the addition of the fluorescent dyes mentioned earlier. Thus, the low recovery rate and the cross-reaction of the method may make the results less reliable.

The third inconvenience of this method is that it ultimately renders little information for the time and the money that it requires. After filtration of up to 100 L of water, concentrating it, and finally analysing the recovered material by microscopy for potentially many hours, the only information it can give is whether structures similar to (oo)cysts have been observed. Because of the possibility of cross-reaction as mentioned earlier, structures with the same shape as the targets are easily misidentified as (oo)cysts, potentially leading to an inaccurate count [53]. Finally, the method is limited to determining the presence/absence of (oo)cysts, and is incapable of producing essential data such as the species of the protozoa, their viability, etc. Therefore, improvements could be made by generating more information from the samples analyzed.

## 3. Review of the Molecular Experiments Targeting *Cryptosporidium* spp. and *Giardia* spp. from Environmental Water Samples

### 3.1. Literature Review Process

With the objective of being as thorough as possible, articles were reviewed from the discovery of either parasite (approximately 1910s for both). The keywords *Cryptosporidium* and *Giardia* were used respectively on Web of Science to find as many articles as possible on either microorganism. A total of 5700 articles were selected for *Cryptosporidium* and 5200 for *Giardia* based on the content of their abstract and the keywords chosen by the authors. Of those, 2300 articles for *Cryptosporidium* and 1600 for *Giardia* were read for their potential interest regarding detection of these parasites from various matrices and/or from different hosts. Finally, 166 articles for *Cryptosporidium* and 111 for *Giardia* were analyzed in greater detail, since these studies included water samples that were processed with biomolecular techniques. Detailed analysis of these articles can be found in Supplementary Material (Table S2).

### 3.2. Description of the Techniques

#### 3.2.1. Pre-Biomolecular Era (Until 1990)

The *Cryptosporidium* genus was first mentioned in 1907 but was more explicitly described in 1910 [19,55]. From the moment this new genus was discovered, it attracted the attention of several research groups who tried to observe it with a variety of approaches such as differential interface contrast microscopy, transmission electronic microscopy and light microscopy coupled with several slide staining methods (ex.: Giemsa, Ziehl-Neelsen, hematoxylin and eosin, periodic acid-Schiff, phloxine tartrazine) [6,56,57,58,59,60]. Most of these studies were centered around the analysis of animal feces, as different groups of researchers raced to discover *Cryptosporidium* oocysts in new host animal species [14,16,20,24,61,62,63]. But it was only in 1983 that it was established that *Cryptosporidium* could indeed infect humans by zoonosis [64]. Cryptosporidiosis was first considered a disease that infected mostly immunocompromised individuals. However, later studies showed that immunocompetent individuals were also susceptible. A few years later, *Cryptosporidium* was officially classified a waterborne parasite just like *Giardia* spp. [65,66]. At this point it was clear that a method was necessary to detect *Cryptosporidium* as well as *Giardia* spp. (oo)cysts from water sources to limit the health risks to consumers. The first step was to develop a diagnostic analysis for water samples based on the fundamental studies performed on animal and human feces. Since *Cryptosporidium* and *Giardia* are difficult to culture and are found at low concentrations in water samples, conventional methods used to monitor fecal or total coliforms could not be applied. In the optic of concentrating (oo)cysts from samples, a process that used either a filtration capsule or a filtration membrane was coupled with microscopy to increase the chances of detection [67,68]. Since conventional staining techniques often lack sensitivity, a new approach was considered: fluorescent and immunofluorescent dyes. Even today, the most common method used today is DAPI and antibodies tagged with FITC. The use of fluorescence increased the strength of the signal emitted and the use of antibodies recognizing specific epitopes on cells allowed a significant increase in specificity.

*Giardia* was discovered much earlier than *Cryptosporidium*, although the first articles still available on Web of Science were published in the 1910s [69]. A well-known anecdote is that Antoni van Leeuwenhoek himself in the process of testing the magnification power of his microscope with his own fecal samples produced a sketch of what was later identified as a vegetative *Giardia* cell [70]. History reveals another epidemic of Giardiasis when soldiers in 1915 came back from two major battlefields, Flanders and Gallipoli (now found in Belgium and Turkey, respectively) with hard-to-treat gastrointestinal symptoms [71,72]. But the pathogenic nature of *Giardia* was finally agreed upon after epidemics exploded in English nurseries in the 1940s that affected young children as well as their caretakers [73,74]. At that time, the only means of diagnosing *Giardia* infection was via the examination of patients’ feces by microscopy as well as some procedures based on staining such as with Gram’s iodine [73,75]. Before being acknowledged as a waterborne agent, it was first recognized as the cause of venereal disease [76,77] and as a pathogen capable of zoonosis [71,78,79]. It was in the 1970s and 1980s that the scientific community concluded that *Giardia* could be transmitted through water [80,81,82]. From then, there were many studies investigating how to eliminate *Giardia* cysts in water [83,84,85,86]. But in these years, the main method for detection of the cysts from water samples remained based on filtration and microscopy [87]. The 1980s marked the beginning of the use of immunofluorescence for the detection of this protozoan, first for clinical samples but later on for water samples as well [88,89,90]. This was soon complemented with other methods such as ELISAs (enzyme-linked immunosorbent assays) and Enzyme-immunoassays (EIA) [91,92,93,94]. It is also important to specify that the U.S. Environmental Protection Agency authorized the first version of a detection method of *Giardia* cysts in water samples in 1976 [95]. However, it was quickly discovered that the performance and cyst recovery of most of these methods, even the U.S. EPA one, were very low [95,96,97]. Improvement was required to ensure reliable data.

#### 3.2.2. Biomolecular Era (From 1990)

Table S2 provides a detailed, yet non-exhaustive, list of research articles using molecular biology to detect, quantify and/or identify species of *Cryptosporidium* and *Giardia* from water samples (environmental water samples, treated water samples, wastewater samples, etc.). This table represents the data that was compiled in the other tables and figures presented below. For simplicity’s sake in the table, complete titles such as the small-subunit gene and 18S rRNA gene were abbreviated to the 18S rRNA gene in the table. Also, because most studies used Sanger technology, the heading ‘’sequencing’’ used in this table refers to this technique. Next generation sequencing is specifically mentioned when it was used. It is important to explain that the limits of detection specified in this table are the ones clearly stated in the article itself. Any limit of detection present in Supplementary data or mentioned in a previous article was not considered and classified as “Data not available” along with the other articles not presenting a limit of detection value.

**Figure 3:**
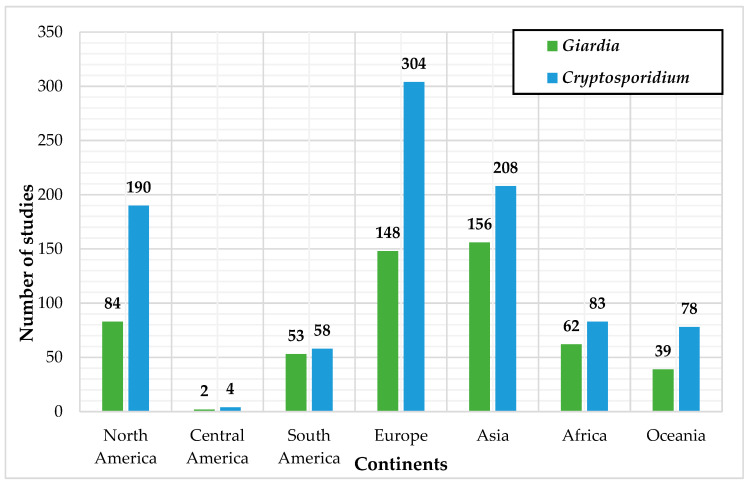
Origin of the water samples from biomolecular studies reviewed according to the parasite of interest.

Also, the origin of the water samples in these biomolecular studies was analyzed and sorted according to the parasite of interest as well as the continent (Figure 3). It was found that samples predominantly came from Europe and Asia, followed by North America. Among the aspects to explain the distribution of these frequencies, outbreaks are likely to be a major investigation trigger (ex.: *Cryptosporidium* waterborne outbreak of Milwaukee, Wisconsin, in 1993 [98] and in Swindon and Oxfordshire, United Kingdom in 1991 [99]). Increasing numbers of HIV/AIDS cases worldwide since the 1980s may also have brought concerns toward parasitosis, since people suffering from this infection are immunocompromised and, therefore, more vulnerable to pathogens [100]. As can be seen in Figure 3, some regions of the world are still underrepresented in the genotyping of these parasites from water samples, particularly Central and South America. Several factors may be responsible for this disparity, such as the high costs relative to molecular biology applications, especially in low-income regions, or the preference to use traditional detection techniques such as microscopy for which expertise was previously developed. Unfortunately, this lack of information may prevent us from getting a much more complete picture of the presence of these parasites and of their genotypes/species throughout the world, especially in underdeveloped regions that are strongly affected by these organisms.

##### Techniques Used

During our literature review, the molecular biology techniques used to detect *Cryptosporidium* and *Giardia* from water samples were extracted and the number of times each was presented in the literature for that purpose was compiled. Also, the highest and lowest limit of detection achieved for each technique was determined. The results of this compilation can be found at Table 1.

The use of molecular biology to detect *Cryptosporidium* and *Giardia* became more common beginning in the 1990s, starting first with classical methods such as DNA hybridization (e.g., Southern Blot), and dot blot. But soon PCR took over as the main means of detection in combination with either gel electrophoresis or membrane-transferring techniques for the separation and visualization of amplified products. This method is based on the annealing of short oligonucleotides called primers that recognize the flanking regions of the sequence targeted for amplification. A DNA polymerase, directed by the primers, binds to the target sequence and uses it as a template to synthesize a copy. This is repeated many times through an exponential process, and the PCR products are then detected by migration on an agarose gel. Variants of this PCR approach were quickly adopted such as nested PCR, semi-nested PCR or RFLP-PCR to improve sensitivity. Nested PCR consists of a succession of two PCR reactions, the first with external primers and the second with internal primers creating a shorter amplicon within the first PCR’s amplification products. Semi-nested PCR is when one of the external primers is reused in the second round of PCR. RFLP-PCR (restriction fragment length polymorphism) is the combination of a PCR protocol with a restriction digestion to cut PCR amplicons into smaller fragments. In several studies, RAPD-PCR (random amplified polymorphic DNA PCR) or AP-PCR (arbitrarily primed PCR) were also used to increase the yield of genetic information. With these techniques, primers are randomly generated among genome sequences of the organism to allow typing based on the pattern obtained by gel electrophoresis. As the years went by, other questions arose, such as whether there were *Cryptosporidium* or *Giardia* present in samples, and if so, how many there were and whether they were still viable. To answer these additional questions, real-time PCR and reverse transcription PCR (mostly the TaqMan option) were widely used. These techniques allow the quantification of a DNA target and the study of gene transcription. Also, as other parasitic organisms were found to cause disease in animals and/or humans, multiplex-PCR was developed, a technique that allows the detection of more than one target at the same time from a single sample. As the final version of the U.S. EPA Method 1623 was published in January 1999, several research studies chose to add an immunomagnetic separation step to the preparation of their samples destined for molecular biology, making it one of the most popular concentration methods used for the detection of *Cryptosporidium* and *Giardia* from water samples. Around the 2000s, DNA sequencing also became a very popular technique to use to gain more information about these protozoa, with a preference for the ABI 3730 technology. Finally, Notomi and collaborators’ developed a Loop-mediated isothermal amplification (LAMP) technique in the year 2000 [103] which became one of the more commonly used methods for the detection of these organisms [104,105,106,107]. This process is also based on DNA amplification mediated by primer annealing, but uses at least two pairs of primers and a DNA polymerase with a strand-displacement activity [103].

##### Most Frequent Genetic Targets

Depending on the objectives of the study, different genetic targets were chosen to detect, quantify or identify *Cryptosporidium* and *Giardia* from water samples (see Table 2 for more details). However, for *Cryptosporidium*, the most common targets were the 18S rRNA gene and genes coding for oocyst cell wall proteins (like the *Cryptosporidium* oocyst wall protein (COWP) gene), Heat-shock protein 70, thrombospondin related adhesive protein genes (TRAP-C1 and TRAP-C2), glycoprotein-60, S-adenosyl-methionine synthetase-1 and the DNA-J-like protein. For *Giardia*, the most common targets for biomolecular techniques were the 18S rRNA gene (see Box 1 below on 16S vs. 18S rRNA gene in *Giardia*) and genes coding for giardins (like the β-giardin gene), triose phosphate isomerase, glutamate dehydrogenase and elongation factor alpha-1.

Box 1 **16S or 18S rRNA gene in *Giardia*: are they the same gene?**

Several articles cited in this review mentioned that they targeted the 16S rRNA gene in *Giardia* such as [48,108,109,110,111,112]. The usage of the two terms, 18S rRNA gene and 16S rRNA gene, raised a questioning about the validity of using either of these names. To solve this issue, we decided to select the sequences available in the NCBI database under the names of 16S rRNA gene and 18S rRNA gene belonging to *Giardia* spp. and to align them bioinformatically with the Clustal Omega software. The alignment was visualised with Jalview and it turned out that these two labels correspond to the same gene in the *Giardia* genus. Therefore, since *Giardia* is a eukaryotic organism, the authors suggest that the 18S rRNA gene label be used for future studies to avoid further confusion.

### 3.3. Pros and Cons of Biomolecular Methods

A synthesized version of these pros and cons is presented at Table S1.

#### 3.3.1. Pros of Biomolecular Methods

Since biomolecular methods target DNA instead of entire cells, it does not require the growth of the organism of interest for detection. This is quite a significant advantage when targeting *Cryptosporidium* and *Giardia*, since both are parasites and therefore require nutrients provided by the host to proliferate. Also, the targeting of nucleic acids does not require the collection of intact or even whole cells to do the analysis, which might be tricky for certain types of detection methods such as those based on microscopy. Molecular methods can thus be relatively more sensitive because both intact (oo)cysts and fragments of (oo)cysts can be detected.

Furthermore, compared to microscopy-based assays, molecular methods are globally more reliable since they do not depend on the skills of the microscopist to distinguish an (oo)cyst from any other cell with a similar appearance. Consequently, the biomolecular process is shorter to do, not only in the preparation of the samples but also for the analysis of the results, which tend to be less subjective than with microscopy.

Finally, the molecular biology techniques have the capacity to push the analysis of these microorganisms a step further by finding complementary information. For example, with appropriate primers, identification to the genus level can be achieved. Also, real-time PCR allows a more precise quantification of the amount of a genetic target, and therefore the organism, in the sample [113,114]. Assessment of the (oo)cyst viability is possible with techniques such as reverse transcription real-time PCR, which allows the determination of whether a target gene is transcribed within the organism or not, which has been shown to correlate with viability [115]. The sequencing of nucleic acids can help identify the species of *Cryptosporidium* and *Giardia* found in a sample, and since not all species are necessarily found in the same type of environment, it can give insight into the source of the contamination of the water by these parasites. The complementary information gained with biomolecular techniques allows research teams and environmental analysis laboratories to get more from their precious samples and to learn more from their study site when compared to microscopy-based approaches.

#### 3.3.2. Cons of Biomolecular Methods

DNA-based approaches also have disadvantages. Because DNA is not only contained in cells but can also be found freely in the environment, every step of the process, from sampling to the acquisition of the results, is susceptible to contamination by external sources of DNA such as the manipulators, the container used, etc. However, *Cryptosporidium* and *Giardia* are generally not among the most abundant microorganisms found in environmental samples, so this source of contamination is likely to be minimal. It is also important to consider that distinguishing DNA coming from a living cell versus DNA from a dead one can be a difficult task to do, which can lead to an overestimation of the risks associated with the presence of these parasites in a sample when using biomolecular methods.

Also, another issue to keep in mind is that since they are eukaryotic cells, just like for the handlers of the samples and other macroorganisms, extreme care must be taken when choosing the genetic targets and designing the primers to avoid amplifying DNA from another source than the two parasites of interest. Some techniques can be quite powerful with very low limits of detection (one or two copies of the target gene) such as LAMP. However, if the primers used have even a small resemblance to contaminant DNA, it can give a positive signal with a strong intensity with background DNA material, which can be misleading. This raises another issue concerning the detection of *Cryptosporidium* and *Giardia* by molecular means, which is the absence of a standardized methodology. Many studies that were done in the past focused on the detection of these organisms (see Table S2). As mentioned earlier, many different targets and primers can be used depending on the ultimate goals of the project. Since every one of them has different specificities, comparing their performance is a difficult, if not impossible, task. The U.S. EPA 1623.1 method has the advantage of being the standard procedure which allows comparison between studies, something biomolecular techniques presently do not do.

Also, several elements can undermine the efficiency of the biomolecular technique used. First, since these organisms’ DNA is not only inside the cells but also contained in rigid (oo)cysts, care must be taken with the cell lysis protocol used. Insufficient cell lysis can lead to less genetic target to amplify by PCR and consequently bias the data [116]. Similarly, nucleic acid amplification techniques tend to be sensitive to the presence of various compounds in environmental samples like humic acids, clay, etc. that act as inhibitors of the amplification process [117,118]. DNA extracts obtained from samples must be as pure as possible to avoid getting false negatives.

Finally, since the detection of *Cryptosporidium* and *Giardia* have historically been done by microscopy and that molecular biology is an ever-evolving domain, many diagnostic laboratories may not possess the instruments required to amplify DNA. Costly purchases might be necessary to implement these techniques, which might raise some concerns, especially since no standardized molecular technique has been agreed upon. Laboratories with fewer resources may not be able to buy these instruments and reagents and might prefer to continue to rely on the microscopic methods for the detection of these parasites. In the long run, biomolecular techniques may be more profitable (money-wise and information-wise), but the transition from one means of detection to the next may be a leap not every laboratory can afford.

## 4. Recommendations on Biomolecular Techniques

Based on the information gathered from the literature, here are some recommendations that we can provide to fellow colleagues facing a dilemma on which technique to choose. Although a perfect method that applies to all scenarios does not exist, it is possible to determine the most appropriate one based on several aspects like the aim of the experiment (presence/absence, quantification, viability assessment, etc.), the concentration of parasites expected in the type of sample (ex.: generally, there are more (oo)cysts in wastewater samples than groundwater samples) and the likelihood of the sample containing PCR inhibitory compounds.

Based on the results from previous studies, the best choice for a presence-absence study seems to be the LAMP method, not only for *Giardia* but for *Cryptosporidium* as well. It has been found to be very efficient in the detection of bacteria, viruses and other eukaryotes [119,120,121]. This technique is documented to be the least sensitive to PCR inhibitory substances. Therefore, it should be favored in situations where few (oo)cysts are expected, like with environmental water samples where the method has been shown to successfully detect the target in as little as femtograms worth of DNA per reaction (see Table 2 for more details). It can also be used in wastewater samples where the concentration of PCR inhibitory compounds is likely to be high, since it is the least sensitive technique to this problem. However, pairing LAMP with fluorescent DNA-intercalating dye can be complex when accurate quantification of the copy number is desired. Therefore, we suggest that LAMP should be used primarily when the determination of presence/absence is the objective.

In a situation where quantification of (oo)cysts is required, quantitative PCR is the standard technique. A lot of variability was noticed among studies relative to the sensitivity obtained. Therefore, we strongly suggest testing different primer pairs, when possible, to select the most sensitive ones. If PCR inhibitors are also an issue in the study context, adding anti-inhibitory compounds like bovine serum albumin, polyvinylpolypyrrolidone (PVPP) or betaine to the samples should be tested. Also, some extra purification steps can be applied to reduce the concentration of inhibitors [118]. New techniques such as droplet digital PCR should also be considered for quantification purposes, but these processes can be quite costly.

When assessing the viability of (oo)cysts is necessary, RNA extraction and a reverse transcription step before a DNA amplification technique such as LAMP or PCR seems to be the best option. It allows the investigator to determine whether a target gene was expressed in the (oo)cysts, indicating that the cell was metabolically active. It is also important to remember that infectiousness and viability are two different concepts, and that as of now, the best technique to determine the infectiousness involves an animal model [108].

## 5. Conclusions

1. *Cryptosporidium* and *Giardia* are parasitic protozoa that can transmit waterborne diseases, especially if not adequately monitored in water sources.
2. The U.S. EPA developed Method 1623.1 to detect these protozoa from water samples. Briefly, this protocol consists of the filtration of 100 L of the sample onto a 1 µm filter, the elution of the biological material from the filter, the concentration of (oo)cysts by immunomagnetic separation and the detection of whole cells by fluorescent microscopy with FITC and DAPI stains.
3. Historically, *Cryptosporidium* and *Giardia* were primarily detected by microscopy until the 1990s, when molecular biology emerged. Since then, a multitude of PCR protocols, PCR variants, real-time techniques, isothermal protocols and sequencing-based protocols were designed to improve the detection and characterization of these protozoa in aquatic samples. These techniques continue to evolve and improve.
4. U.S. EPA Method 1623.1 and biomolecular techniques both have specific advantages and limitations that must be taken into consideration with the objectives of the study before deciding which method is most appropriate to use.
5. The present review article aims to provide useful insights and perhaps even trigger new ideas, for researchers, drinking water managers, epidemiologists, and public health specialists for the improvement of the monitoring of *Cryptosporidium* and *Giardia* spp. in water sources.

## Figures and Tables

**Table 1:** **Frequency of use of different biomolecular techniques and detection limits achieved.** To compare the detection limits retrieved from the literature, the values were converted mathematically into (oo)cysts per microliter. When the detection limit was given in weight instead of in cells, the reference values of 40 fg of DNA per oocyst and of 313 fg of DNA per cyst were used for the conversion, as stated in [101,102]. Also, since *Giardia* is a polyploid organism and thus the number of genome copies varies between cells, one genome copy per nucleus (four copies per cyst) was assumed to simplify the conversion. Finally, to standardize the limits of detection, the volume of the reaction was systematically assumed to be of 1 µL for more simplicity.

Organism	Technique	Frequency	Lower Detection Limit	Higher Detection Limit
*Giardia* spp.	DNA hybridization	5	1–5 cysts/mL	1000 copies/reaction
	PCR and derivatives	81	10 cysts/100 L	100 cysts/reaction
	Real-time PCR	27	5 cysts/L	50 cysts/reaction
	LAMP	3	100 fg of target DNA/mL	100 fg of target DNA/mL
*Cryptosporidium* spp.	DNA hybridization	6	Non applicable	1000 copies/reaction
	PCR and derivatives	139	1–5 oocysts/20 L	0.13 ng of DNA per mL
	Real-time PCR	31	10 oocysts/100 L	50 oocysts/reaction
	LAMP	4	100 fg of target DNA/mL	1.8 fg/reaction

**Table 2:** Frequency of use of different genetic targets by molecular biology studies.

Organism	Genetic Target	Time Used
*Giardia* spp.	Giardin gene (ex. β-giardin)	49
	18S rRNA gene	39
	Glutamate dehydrogenase gene	34
	Triose phosphate isomerase gene	31
	Elongation factor gene (ex.EF1-α)	4
	Heat-shock gene	2
*Cryptosporidium* spp.	18S rRNA gene	122
	Glycoprotein-60 gene	26
	Oocyst cell wall protein gene	25
	Heat-shock protein gene	19
	Other genes	11
	Uncharacterized genomic sequences	9
	TRAP-C genes	3
	S-adenosyl-methionine synthetase-1 gene	3

## Data Availability

Not applicable.

## Supplementary Materials

The following supporting information can be downloaded at: https://www.mdpi.com/article/10.3390/microorganisms10061175/s1, Table S1. Recapitulation of the pros and cons of the techniques used to detect *Cryptosporidium* spp. and *Giardia* spp. from water samples; Table S2: Complete description of biomolecular studies targeting *Cryptosporidium* spp. and *Giardia* spp. in water samples. (The [122,123,124,125,126,127,128,129,130,131,132,133,134,135,136,137,138,139,140,141,142,143,144,145,146,147,148,149,150,151,152,153,154,155,156,157,158,159,160,161,162,163,164,165,166,167,168,169,170,171,172,173,174,175,176,177,178,179,180,181,182,183,184,185,186,187,188,189,190,191,192,193,194,195,196,197,198,199,200,201,202,203,204,205,206,207,208,209,210,211,212,213,214,215,216,217,218,219,220,221,222,223,224,225,226,227,228,229,230,231,232,233,234,235,236,237,238,239,240,241,242,243,244,245,246,247,248,249,250,251,252,253,254,255,256,257,258,259,260,261,262,263,264,265,266,267,268,269,270,271,272,273,274,275,276,277,278,279,280,281,282,283,284,285,286,287,288,289,290,291,292,293,294,295,296,297,298,299,300,301,302,303,304,305,306,307] are cited only in supplementary materials but are part of the literature review made for this publication).
