# Supplementary material for: Detection of Cryptosporidium spp. and Giardia spp. in Environmental Water Samples: A Journey into the Past and New Perspectives
Source: Microorganisms. 2022 Jun 7;10(6):1175. doi: 10.3390/microorganisms10061175 (PMC9228427; doi:10.3390/microorganisms10061175)
Supplement: Supplementary file 1 [file microorganisms-10-01175-s001.zip › microorganisms-1763038-supplementary.pdf]

Supplementary materials

This supplementary material section contains two tables: S1 (p.1) and S2 (p.2)

Table S1. Recapitulation of the pros and cons of the techniques used to detect *Cryptosporidium* spp. and *Giardia* spp. from water samples

Technique	Pros	Cons
US.EPA Method 1623.1	• Possibility of concentrating large volumes of water (up to 100 L),<br>• Detection limit of one oocyst per 100 L,<br>• No PCR amplification biases,<br>• Simultaneous use of several fluorescent for more confidence in the identification,<br>• Quantification (enumeration) possible.	• Time consuming and high costs associated with this analysis,<br>• Low recovery and possibility of cross-reaction,<br>• Requirement of intact parasitic cells for identification,<br>• Capable of giving few information about these parasites (ex.: no species identification, no viability assessment),<br>• Identification biased by the skills of the microscopist.
Biomolecular methods	• No growth of microorganisms required,<br>• No intact cells of the parasites required,<br>• Identification not biased by the skills of the manipulator (less subjective),<br>• Capable of giving complementary information about these parasites according to the technique chosen (ex.: species identification, viability assessment).	• Susceptible to contamination by external sources of DNA,<br>• Not distinguishing DNA from live or dead cells,<br>• Depending on primers, susceptible to DNA from other eukaryotes,<br>• Susceptible to the efficiency of the cell lysis method chosen,<br>• Susceptible to PCR inhibitory substances,<br>• No standardized protocol and not accessible to all laboratories.

Table S2. Complete description of biomolecular studies targeting *Cryptosporidium* spp. and *Giardia* spp. in water samples

Organism(s) of interest	Technique used	Gene targeted	Type of sample	Detection limit	Reference
Giardia spp.	Hybridization with a cDNA probe on DNA extract and autoradiography	16S rRNA gene (?)	Purified cells, treated sewage and river water samples	1-5 cysts/mL	[1]
Giardia spp.	PCR, Multiplex PCR, gel electrophoresis, Southern Blot and oligonucleotide hybridization	Giardin gene	Purified cysts, spiked environmental water samples	1 cyst/reaction	[2]
Cryptosporidium spp.	DNA hybridization assay and PCR	18S rRNA gene	Environmental water samples	20-100 oocysts/reaction	[3]
Cryptosporidium spp.	PCR, Dot blot and gel electrophoresis	18S rRNA gene	Cow fecal preparations, spiked environmental water samples, wastewater samples	1-200 oocysts/reaction (depending on the level of purity of the sample)	[4]
Cryptosporidium parvum	PCR on DNA extract and gel electrophoresis or DNA hybridization	Gene fragment CpR1 (an oocyst protein gene)	Spiked raw milk samples, spiked water samples, purified oocysts	10 oocysts per 20 mL of raw milk	[5]
Cryptosporidium sp. and *Giardia sp.*.	PCR, nested-PCR on DNA extract, gel electrophoresis and Southern Blot	*Cryptosporidium*: Oocyst cell wall protein (Nested PCR)<br><br>*Giardia*: Giardin gene (PCR)	Sewage samples, purified (oo)cysts	10^2 oocysts/L	[6]
Cryptosporidium parvum	Extraction of HSP70 mRNA, RT-PCR, PCR, gel electrophoresis and Southern Blot	Heat-shock protein 70 (HSP70)	Oocysts purified from feces, environmental water samples,	1 oocyst/reaction	[7]

Giardia sp. and *Cryptosporidium* sp.	PFGE, PCR, recombinant probe hybridization and DNA hybridization	16S rRNA gene (?)	Raw water samples, treated drinking water samples and raw sewage samples	10 (oo)cysts/100 L	[8]
Cryptosporidium parvum and *Giardia lamblia*	UDP-inactivation, PCR, Multiplex-PCR, gel electrophoresis, DNA hybridization and enzymatic digestion	*Cryptosporidium*: 18S rRNA gene and two uncharacterized genomic DNA targets<br><br>*Giardia*: An HSP gene, giardin gene, 18S rRNA gene	Purified (oo)cysts, environmental water samples	1-100 (oo)cysts/reaction (depending on the sample quality)	(Rochelle et al., 1997a)
Cryptosporidium parvum	PCR, Multiplex PCR, RT-PCR, oligonucleotide hybridization and Southern Blot	Heat-shock protein 70 (HSP70)	Cultured *Cryptosporidium* cells, environmental water samples	1-10 oocysts (environmental samples) per reaction<br><br>1 oocyst (purified cells) per reaction	(Rochelle et al., 1997b)
Cryptosporidium parvum	PCR on DNA extract	An oocyst wall protein gene, two uncharacterized genomic DNA target and 18S rRNA gene	Purified oocysts, environmental water samples	< 10 oocysts (purified oocysts) per reaction<br><br>100 oocysts (spiked environmental water samples) per reaction	[10]
Cryptosporidium parvum	PCR, ELISA-based PCR assay (Digene SHARP Signal System assay), Nested-PCR and gel electrophoresis	CpR1 gene	Purified oocysts, spiked municipal water samples, environmental water samples	1-10 oocysts/reaction	[11]
Cryptosporidium parvum	Immunomagnetic separation, RAPD-PCR and gel electrophoresis	Not applicable	Purified oocysts, environmental water samples	$3x10^3$-$1x10^4$ oocysts/reaction	[12]
Cryptosporidium spp.	PCR, Nested-PCR, AP-PCR on DNA extract and gel electrophoresis	18S rRNA gene (PCR and Nested-PCR)	Purified oocysts, spiked backwash water sample	0,13-4,22 ng of DNA per mL (AP-PCR)	[13]

		Non applicable (AP-PCR)		0,00405-0,13 ng of DNA per mL (Nested-PCR)	
Cryptosporidium sp. and *Giardia sp.*	Reverse transcription RT-PCR, Multiplex-PCR and gel electrophoresis	*Cryptosporidium*: HSP70 gene<br><br>*Giardia*: Giardin gene, *Giardia* heat shock gene	Purified oocysts, environmental water samples (spiked or not)	1 (oo)cyst/reaction	[14]
Cryptosporidium parvum	Competitive quantitative-PCR, PCR and hybridization	An oocyst wall protein gene	Purified oocysts, spiked environmental water samples	10^3 oocysts/100 L of environmental water	[15]
Cryptosporidium parvum	Immunomagnetic separation, CC-PCR, gel electrophoresis, cloning and sequencing	HSP70 gene	Purified oocysts, raw water and filter backwash water samples spiked or not	Data not available	[16]
Cryptosporidium parvum	Immunomagnetic separation, PCR, Nested-PCR, gel electrophoresis and dot blot hybridization	Uncharacterized genomic DNA target	Purified oocysts, spiked tap water	10 fg of DNA (approx.. 1 genome)	[17]
Cryptosporidium parvum	Immunomagnetic separation, Nested-PCR and gel electrophoresis	An oocyst wall protein gene	Purified oocysts, human fecal samples, animal fecal samples and water samples	1000 oocysts/g of feces and 100 oocysts/mL of water	[18]
Cryptosporidium parvum	Immunomagnetic separation, PCR, gel electrophoresis and dotblot hybridization,	Uncharacterized genomic DNA target	Purified oocysts, river water samples spiked or not, tap and raw water samples	1-5 oocysts/20 L of water sample	[19]
Cryptosporidium sp.	Immunomagnetic separation, RFLP-PCR and gel electrophoresis	18S rRNA gene	Purified oocysts and environmental water samples	10 oocysts/10 mL of purified water sample	[20]
Giardia duodenalis	RFLP-PCR and pulse-field gel electrophoresis (PFGE)	Triose phosphate isomerase gene (tpi)	Human, animal and environmental water samples, purified cells	Data not available	[21]

Cryptosporidium parvum	PCR, qPCR and gel electrophoresis	18S rRNA gene	Raw water samples (spiked or not) and sludge samples	Data not available	[22]
Cryptosporidium spp.	Immunomagnetic separation, RFLP-Nested-PCR and sequencing	18S rRNA gene	Surface water samples	Data not available	[23]
Cryptosporidium parvum	Immunomagnetic separation, PCR, gel electrophoresis and sequencing	18S rRNA gene and TRAP-C2 gene	Surface water samples	0,09 oocysts/10 L	[24]
Cryptosporidium spp.	Immunomagnetic separation, RFLP-PCR, gel electrophoresis and sequencing	18S rRNA gene and TRAP-C2 gene	Environmental water samples and mussel samples	10 oocysts/10 mL of environmental water sample	[25]
Cryptosporidium parvum	Immunomagnetic separation, RFLP-PCR and gel electrophoresis	Poly-threonine locus	River water samples	Data not available	[26]
Giardia spp.	Immunomagnetic separation, RFLP-PCR, gel electrophoresis	Glutamate dehydrogenase gene	Purified oocysts and sewage sludge samples	625 cysts/mL	[27]
Cryptosporidium spp.	Immunomagnetic separation, RFLP-Nested PCR, gel electrophoresis and sequencing	18S rRNA gene	Surface water and wastewater samples	Data not available	[28]
Giardia sp. and *Cryptosporidium* sp.	RFLP-Nested-PCR and gel electrophoresis	18S rRNA gene	Animal fecal samples and sewage water samples	Data not available	[29]
Cryptosporidium sp.	Immunomagnetic separation, RFLP-Nested-PCR, cloning in a plasmidic vector and sequencing	18S rRNA gene	Purified oocysts, surface water samples in Massachusetts, fecal samples	1 oocyst per reaction	[30]
Cryptosporidium sp. and *Giardia sp.*	Immunomagnetic separation, PCR, gel electrophoresis and Southern hybridization	*Cryptosporidium*: COWP gene and non-characterized target locus<br>*Giardia*: Glutamate dehydrogenase gene	Surface water samples (spiked or not)	50-100 oocysts and 50 cysts	[31]

Cryptosporidium parvum	Immunomagnetic separation, RFLP-Nested-PCR and gel electrophoresis	18S rRNA gene	Purified oocysts, environmental water samples from Colorado	5 oocysts/reaction	[32]
Giardia sp.	RFLP-PCR, gel electrophoresis, cloning in a plasmidic vector and sequencing	16S rRNA gene	Environmental water samples and sewage samples	Data not available	[33]
Cryptosporidium sp.	PCR, Nested-PCR, gel electrophoresis and sequencing	18S rRNA gene	Purified oocysts, environmental water samples from Europe	1 oocyst/reaction	[34]
Cryptosporidium sp. and *Giardia sp.*	RFLP-PCR, gel electrophoresis and sequencing	*Cryptosporidium:* 18S rRNA gene<br><br>*Giardia*: β-giardin gene	Wastewater samples	Data not available	[35]
Cryptosporidium parvum	Immunomagnetic separation, PCR, RT-PCR and gel electrophoresis	HSP70 gene	Purified oocysts and spiked tap water	10 oocysts/100 L of water	[36]
Cryptosporidium parvum	Immunomagnetic separation, PCR, Nested-PCR,	Cp41 gene, TRAP-C1 gene	Purified oocysts, spiked water samples	Data not available	[37]
Cryptosporidium spp.	Immunomagnetic separation, Cell-culture-PCR, gel electrophoresis, cell culture quantitative sequence detection and sequencing	HSP70 gene	Environmental water samples	Data not available	[38]
Cryptosporidium sp.	Immunomagnetic separation, RFLP-Nested PCR, gel electrophoresis and sequencing	18S rRNA gene	Reclaimed water samples and spiked tap water samples	5-10 oocysts per reaction	[39]
Cryptosporidium parvum	Nested-PCR and microcapillary electrophoresis	18S rRNA gene	Purified oocysts, secondary effluent samples from wastewater treatment plants	11-4200 oocysts/L of sample	[40]

Cryptosporidium sp. and *Giardia sp.*	PCR (*Cryptosporidium*), RAPD-PCR (*Giardia*), AP-PCR (*Giardia*) and gel electrophoresis	*Cryptosporidium*: Uncharacterized genomic sequences, CpR1 gene, 18S rRNA gene and HSP70 gene (PCR)<br><br>*Giardia*: Non-applicable	Water and sediment samples	Data not available	[41]
Giardia spp.	TaqMan qPCR	Elongation factor 1A gene	Purified cysts and sewage samples	0,45 cysts per reaction	[42]
Cryptosporidium spp. and *Giardia spp.*	Immunomagnetic separation and PCR	Non-specified (*Cryptosporidium*)<br><br>Glutamate dehydrogenase gene (*Giardia*)	Environmental water samples	Data not available	[43]
Giardia duodenalis	Immunomagnetic separation, Nested-PCR and sequencing	Triose phosphate isomerase gene	Wastewater samples	Data not available	[44]
Cryptosporidium sp. and *Giardia sp.*	Immunomagnetic separation, RFLP-PCR and gel electrophoresis	*Cryptosporidium:* COWP gene (RFLP-PCR)<br><br>*Giardia*: Glutamate dehydrogenase gene (PCR)	Wastewater samples, river water samples and mussel samples	Data not available	[45]
Cryptosporidium sp.	Nested-PCR and gel electrophoresis	18S rRNA gene	Stormwater samples and wastewater samples (spiked or not)	5-50 oocysts	[46]
Cryptosporidium spp.	Immunomagnetic separation, Nested-PCR and sequencing	18S rRNA gene and HSP70	Surface water samples and animal fecal samples	Data not available	[47]
Cryptosporidium parvum	Immunomagnetic separation, most probable number-PCR and gel electrophoresis	HSP70 gene	Purified oocysts, raw water samples spiked or not	10^3 oocysts per reaction	[48]

Cryptosporidium spp.	Immunomagnetic separation, PCR, semi-nested PCR, gel electrophoresis and sequencing	18S rRNA gene	Purified oocysts and sewage samples	1 oocyst per reaction	[49]
Cryptosporidium spp.	Immunomagnetic separation, semi-nested PCR, gel electrophoresis and sequencing	18S rRNA gene	Purified oocysts, sewage samples and river water samples	1 oocyst per reaction	[50]
Cryptosporidium spp. and *Giardia* spp.	Semi-nested PCR (*Cryptosporidium*), nested-PCR (*Giardia*), gel electrophoresis and sequencing	*Cryptosporidium*: COWP gene<br><br>*Giardia*: 18S rRNA gene	Wastewater samples	Data not available	[51]
Cryptosporidium spp.	Immunomagnetic separation, Q-probe PCR, Nested-PCR, DGGE migration and sequencing	18S rRNA gene	Purified oocysts and river water samples	0,83 oocyst per sample	[52]
Cryptosporidium sp. and *Giardia sp.*	Immunomagnetic separation, PCR, gel electrophoresis and sequencing	*Cryptosporidium:* COWP gene<br><br>*Giardia*: β-giardin gene, glutamate dehydrogenase gene and 18S rRNA gene	Sewage samples	100 (oo)cysts/ L	[53]
Giardia sp.	Immunomagnetic separation, PCR, gel electrophoresis and sequencing	β-giardin gene, glutamate dehydrogenase gene and triose phosphate isomerase gene	Human fecal samples, sewage samples, soil samples and water samples (treated and raw)	Data not available	[54]
Cryptosporidium sp.	Immunomagnetic separation, RFLP-Nested-PCR, gel electrophoresis and sequencing	18S rRNA gene	Storm water samples	Data not available	[55]
Cryptosporidium spp. and *Giardia spp.*	Immunomagnetic separation, Real-time PCR, Multiplex PCR and gel electrophoresis	*Cryptosporidium*: COWP gene (RT-PCR) and an undefined genomic DNA sequence (Multiplex-PCR)	Purified oocysts, wastewater samples (spiked or not)	50 (oo)cysts per reaction	[56]

		Giardia: β-giardin gene (RT-PCR) and 18S rRNA gene (Multiplex PCR)			
Giardia spp. (Assemblages A, B and E)	PCR, Real-time PCR, gel electrophoresis and sequencing	Triose phosphate isomerase gene	Purified cysts, animal and human fecal samples, wastewater samples	180-250 cysts per L of wastewater (depending on the assemblage)	[57]
Giardia lamblia	Real-time PCR, gel electrophoresis and sequencing	β-giardin gene	Roof-harvested rainwater samples	7-10 gene copies per reaction	[58]
Giardia spp. and *Cryptosporidium spp.*	Immunomagnetic separation, semi-nested-PCR (*Giardia*), nested-PCR (*Cryptosporidium*), gel electrophoresis and sequencing	*Giardia*: β-giardin gene<br><br>*Cryptosporidium*: 18S rRNA gene	Environmental water samples and treated water samples	Data not available	[59]
Cryptosporidium sp.	Immunomagnetic separation, RFLP-Nested PCR, gel electrophoresis and sequencing	18S rRNA gene	Lake water samples	5-10 oocysts/suspension	[60]
Cryptosporidium spp.	Immunomagnetic separation, semi-nested PCR, nested-PCR, gel electrophoresis and sequencing	*Cryptosporidium:* 18S rRNA gene (nested PCR)<br><br>*Giardia*: glutamate dehydrogenase gene (semi-nested PCR) and 18S rRNA gene (nested-PCR)	Surface water samples and sewage samples	Data not available	[61]
Cryptosporidium spp.	Immunomagnetic separation, RFLP-Nested PCR, gel electrophoresis and sequencing	18S rRNA gene	River water samples	Data not available	[62]
Cryptosporidium spp and *Giardia* spp.	Immunomagnetic separation, RFLP-Nested PCR, semi-nested PCR, gel electrophoresis and sequencing	*Cryptosporidium*: 18S rRNA gene (RFLP-nested PCR)	Bovine fecal samples and environmental water samples	Data not available	[63]

		Giardia: β-giardin gene (semi-nested PCR)			
Cryptosporidium spp	Nested-PCR, RFLP-Nested PCR and sequencing	Gp60 gene (Nested-PCR) and18S rRNA gene (RFLP-Nested PCR)	Wastewater samples	1-5 copies of the target DNA per reaction	[64]
Cryptosporidium parvum	Immunomagnetic separation, Reverse transcription-Loop-mediated isothermal amplification and gel electrophoresis	18S rRNA gene	Purified oocysts, Surface and ground water samples	$6x10^{-3}$ oocysts per reaction	[65]
Cryptosporidium spp. and *Giardia spp.*	Immunomagnetic separation, RFLP-PCR, Nested-PCR, gel electrophoresis and sequencing	*Cryptosporidium*: 18S rRNA gene and gp60 gene (RFLP-PCR)<br><br>*Giardia*: β-giardin gene	Environmental water samples	Data not available	[66]
Giardia duodenalis	Immunomagnetic separation, Real-time PCR TaqMan, RFLP-PCR, gel electrophoresis and sequencing	β-giardin gene	Wastewater samples	1 cyst per reaction	[67]
Cryptosporidium spp. and *Giardia duodenalis*	Immunomagnetic separation, Nested-PCR, gel electrophoresis and sequencing	*Cryptosporidium*: 18S rRNA gene (nested PCR)<br><br>*Giardia*: β-giardin gene (semi-nested PCR)	River water samples	100 (oo)cysts per reaction	[68]
Cryptosporidium spp. and *Giardia duodenalis*	Immunomagnetic separation, semi-nested PCR, nested-PCR and gel electrophoresis	*Cryptosporidium:* 18S rRNA gene (nested PCR)<br><br>*Giardia*: β-giardin gene (semi-nested PCR)	Environmental water samples	50 (oo)cysts per reaction	[69]
Giardia spp.	Nested-PCR and sequencing	Triose phosphate isomerase gene	Urban stream water samples	Data not available	[70]

Cryptosporidium spp. and *Giardia spp.*	Immunomagnetic separation, RFLP-Nested PCR, RFLP-PCR and gel electrophoresis	*Cryptosporidium*: 18S rRNA gene (RFLP-Nested PCR)<br><br>*Giardia*: β-giardin gene (RFLP-PCR)	Wastewater samples and treated water samples	Data not available	[71]
Cryptosporidium spp.	Immunomagnetic separation, RFLP-PCR, gel electrophoresis and sequencing	18S rRNA gene	Swine lagoon samples	Data not available	[72]
Cryptosporidium spp. and *Giardia spp.*	Immunomagnetic separation, RFLP-Nested PCR, nested PCR, gel electrophoresis and sequencing	*Cryptosporidium*: 18S rRNA gene and GP60 gene (RFLP-Nested PCR)<br><br>*Giardia*: Triose phosphate isomerase gene (nested PCR)	Wastewater samples and treated water samples	Data not available	[73]
Cryptosporidium sp. and *Giardia sp.*	PCR, Real-time PCR TaqMan and DNA microarray hybridization	18S rRNA gene (both), COWP gene (*Cryptosporidium*) and β-giardin gene (*Giardia*)	Purified DNA, wastewater samples	$1x10^3$ copies of target DNA per reaction (DNA hybridization)<br><br>1-10 oocysts per reaction (PCR)<br><br>100 copies of target DNA per reaction (qPCR)	[74]
Cryptosporidium spp.	RFLP-Nested PCR and gel electrophoresis	18S rRNA gene	Environmental water samples	Data not available	[75]
Cryptosporidium spp.	Immunomagnetic separation, RFLP-Nested-PCR, gel electrophoresis and sequencing	18S rRNA gene	Raw water samples and drinking water samples	Data not available	[76]

Cryptosporidium spp.	Nested-PCR and gel electrophoresis	18S rRNA gene and an uncharacterized genomic locus	Environmental water samples	Data not available	[77]
Cryptosporidium spp. and *Giardia spp.*	Immunomagnetic separation, Real-time qPCR TaqMan	*Cryptosporidium*: COWP gene<br><br>*Giardia*: β-giardin gene	Purified oocysts and sewage samples	1,65 oocysts per reaction and 0,32 cysts per reaction	[78]
Cryptosporidium spp.	Nested-PCR, gel electrophoresis, cloning and sequencing	18S rRNA gene	Recreational and surface water samples	Data not available	[79]
Cryptosporidium spp.	Immunomagnetic separation, RFLP-Nested PCR and sequencing	18S rRNA gene	Environmental and tap water samples	Data not available	[80]
Giardia sp.	Nested-PCR, gel electrophoresis, cloning and sequencing	Glutamate dehydrogenase gene	Surface, raw wastewater and treatment water samples	Data not available	[81]
Giardia lamblia and *Cryptosporidium parvum*	Immunomagnetic separation and qPCR	HSP70 gene (*Cryptosporidium*) and β-giardin gene (*Giardia*)	Environmental and treated water samples	1-10 (oo)cysts per 100 µL of extract	[82]
Cryptosporidium spp.	Nested-PCR, Loop-mediated isothermal amplification and gel electrophoresis	18S rRNA gene (Nested-PCR) and SAM-1 gene (LAMP)	River and tap water samples (spiked or not)	Nested-PCR: 100 fg of DNA per reaction<br><br>LAMP: 1,8 fg of DNA per reaction	[83]
Cryptosporidium spp. and *Giardia duodenalis*	Nested-PCR and sequencing	*Cryptosporidium*: 18S rRNA gene<br><br>*Giardia*: Triose phosphate isomerase gene	Wastewater samples	Data not available	[84]

Cryptosporidium spp. and *Giardia spp.*	Semi-nested PCR, nested-PCR and gel electrophoresis	*Cryptosporidium*: 18S rRNA gene (nested PCR)<br><br>*Giardia*: Glutamate dehydrogenase gene (Semi-nested PCR)	Human and animal fecal samples, environmental water samples	50 oocysts per reaction	[85]
Cryptosporidium sp.	PCR, gel electrophoresis and sequencing	18S rRNA gene	Raw and treated wastewater samples	Data not available	[86]
Giardia duodenalis and *Cryptosporidium spp.*	Nested PCR, gel electrophoresis and sequencing	18S rRNA gene and gp60 gene (*Cryptosporidium*)<br><br>Triose phosphate isomerase gene (*Giardia duodenalis*)	Wastewater, treated wastewater and sludge samples	Data not available	[87]
Cryptosporidium sp. and *Giardia sp.*	Immunomagnetic separation, nested-PCR, gel electrophoresis and sequencing	*Giardia*: 18S rRNA gene and β-giardin gene<br><br>*Cryptosporidium*: HSP70 gene and 18S rRNA gene	Surface and ground water samples, bovine fecal samples	Data not available	[88]
Cryptosporidium sp. and *Giardia sp.*	qPCR	*Cryptosporidium*: COWP gene<br><br>*Giardia*: β-giardin	Wastewater samples	156 cysts and 1,587 oocysts/ml	[89]
Cryptosporidium spp.	Immunomagnetic separation, nested-PCR, gel electrophoresis and sequencing	18S rRNA gene	Drinking water samples	8 oocysts per reaction	[90]
Giardia intestinalis	Immunomagnetic separation, semi-nested-PCR, Reverse transcription PCR, gel electrophoresis and sequencing	Glutamate dehydrogenase gene	Wastewater, river and tap water samples	Data not available	[91]

Giardia sp. and *Cryptosporidium sp.*	Immunomagnetic separation and qPCR	β-giardin gene *(Giardia*) and COWP gene (*Cryptosporidium*)	Environmental water samples and vegetable rinse water samples	40 (oo)cysts per reaction	[92]
Cryptosporidium sp.	Immunomagnetic separation, alternately binding probe competitive reverse transcription PCR (ABC-RT-PCR) and reverse-transcription real-time PCR	18S rRNA gene	River water samples	Data not available	[93]
Giardia duodenalis and *Cryptosporidium* spp.	RFLP-Nested PCR, nested PCR, gel electrophoresis and sequencing	*Cryptosporidium*: 18S rRNA gene (RFLP-Nested PCR), gp60 gene (Nested PCR)<br><br>*Giardia*: Triose phosphate isomerase gene (Nested PCR)	Wastewater samples	Data not available	[94]
Cryptosporidium sp.	qPCR TaqMan, reverse transcription qPCR TaqMan and gel electrophoresis	HSP70 gene	Purified oocysts, surface water samples (spiked or not)	Data not available	[95]
Cryptosporidium spp.	qPCR and sequencing	18S rRNA gene	Recreational and environmental water samples	Data not available	[96]
Cryptosporidium parvum	qPCR	COWP gene	Supply water and wastewater samples	Data not available	[97]
Cryptosporidium sp.	RFLP-Nested PCR, gel electrophoresis and sequencing	18S rRNA gene	Environmental water samples	Data not available	[98]
Cryptosporidium sp.	Immunomagnetic separation, nested-PCR and sequencing	18S rRNA gene	Environmental water samples	Data not available	[99]
Cryptosporidium spp.	Nested-PCR, gel electrophoresis and sequencing	18S rRNA gene	Wastewater and river water samples	Data not available	[100]

Cryptosporidium spp.	Immunomagnetic separation, RFLP-nested-PCR, gel electrophoresis and sequencing	18S rRNA gene	Environmental and treated water samples	Data not available	[101]
Cryptosporidium spp. and *Giardia* spp.	Immunomagnetic separation, replicate RFLP-nested PCR (*Cryptosporidium*), semi-nested PCR (*Giardia*) and sequencing	*Cryptosporidium*: 18S rRNA gene<br><br>*Giardia*: 18S rRNA gene	Raw water samples and wastewater samples	Data not available	[102]
Cryptosporidium spp.	Nested-PCR, loop-mediated isothermal amplification, gel electrophoresis and sequencing	18S rRNA gene (nested PCR) and SAM-1 gene (LAMP)	Sea and tap water samples	100 fg of DNA per reaction (Nested PCR) and 1,8 fg of DNA per reaction (LAMP)	[83]
Giardia sp. and *Cryptosporidium sp.*	Semi-nested PCR (*Giardia*), nested-PCR (*Cryptosporidium*), loop-mediated isothermal amplification and gel electrophoresis	*Cryptosporidium*: 18S rRNA gene (nested-PCR) and SAM-1 gene (LAMP)<br><br>*Giardia*: glutamate dehydrogenase gene (semi-nested PCR) and EF-α gene (LAMP)	Surface water samples	Data not available	[103]
Cryptosporidium spp.	PCR, nested-PCR and gel electrophoresis	18S rRNA gene	Purified oocysts, raw water samples (spiked or not) and sludge samples	2-5 oocysts per reaction	[104]
Cryptosporidium spp.	Immunomagnetic separation, repetitive RFLP-nested PCR, gel electrophoresis and sequencing	18S rRNA gene	Environmental water samples	4 oocysts per sample	[105]
Cryptosporidium spp. and *Giardia lamblia*	Immunomagnetic separation, semi-nested PCR (*Giardia*), nested-PCR (*Cryptosporidium*), gel electrophoresis and real-time PCR	*Cryptosporidium*: 18S rRNA gene and COWP gene<br><br>*Giardia*: β-giardin gene	Wastewater samples	Data not available	[106]

Cryptosporidium spp.	Immunomagnetic separation, qPCR TaqMan and sequencing	DNA-J like protein gene and NTF2 gene	Purified oocysts and river water samples	1-10 oocysts per reaction (depending on the primer pair)	[107]
Cryptosporidium spp.	Immunomagnetic separation, replicate RFLP-Nested PCR and sequencing	18S rRNA gene	Surface water samples	Data not available	[108]
Cryptosporidium spp. and *Giardia spp.*	Immunomagnetic separation, nested-PCR, gel electrophoresis and sequencing	18S rRNA gene	Surface water and wastewater samples	Data not available	[109]
Giardia spp. and *Cryptosporidium spp.*	Immunomagnetic separation, PMA-qPCR	*Giardia*: β-giardin gene, triose phosphate isomerase gene and glutamate dehydrogenase gene<br><br>*Cryptosporidium*: COWP gene	Wastewater samples (spiked or not)	10^3 (oo)cysts/L	[110]
Cryptosporidium sp. and *Giardia sp.*	Nested PCR and sequencing	*Cryptosporidium*: 18S rRNA gene<br><br>*Giardia*: Triose phosphate isomerase gene	Environmental water samples	Data not available	[111]
Cryptosporidium sp. and *Giardia* sp.	PCR, semi-nested PCR, nested-PCR, gel electrophoresis and sequencing	18S rRNA gene	Rainwater samples	Data not available	[112]
Cryptosporidium spp.	Immunomagnetic separation, RFLP-nested-PCR, gel electrophoresis and sequencing	18S rRNA gene	Surface water samples and groundwater samples	Data not available	[113]
Giardia spp.	Immunomagnetic separation, PCR, nested-PCR, gel electrophoresis and sequencing	β-giardin gene (PCR), triose phosphate isomerase gene (nested-	Environmental water samples, wastewater	Data not available	[114]

		PCR) and glutamate dehydrogenase gene (nested-PCR)	samples and fecal samples (humans and animals)		
Cryptosporidium spp.	Immunomagnetic separation, RFLP-nested PCR, gel electrophoresis and sequencing	18S rRNA gene and gp60 gene	Drinking water, wastewater and recreational water samples	Data not available	[115]
Cryptosporidium spp.	Nested-PCR, gel electrophoresis and sequencing	18S rRNA gene and gp60 gene	River water samples	Data not available	[116]
Cryptosporidium spp. and *Giardia spp.*	Immunomagnetic separation, nested-PCR, semi-nested PCR, gel electrophoresis and sequencing	*Cryptosporidium*: 18S rRNA gene (nested-PCR)<br><br>*Giardia*: glutamate dehydrogenase gene (semi-nested PCR)	Wastewater samples	Data not available	[117]
Cryptosporidium sp. and *Giardia sp.*	Nested PCR, semi-nested PCR and gel electrophoresis	*Cryptosporidium*: 18S rRNA gene (nested PCR)<br><br>*Giardia*: glutamate dehydrogenase gene (semi-nested PCR)	Environmental water samples and animal fecal samples	Data not available	[118]
Cryptosporidium sp. and *Giardia sp.*	qPCR, gel electrophoresis and sequencing	*Cryptosporidium*: COWP gene<br><br>*Giardia*: β-giardin gene	River water samples and wastewater samples (both spiked or not)	5 (oo)cysts/L	[119]
Cryptosporidium spp. and *Giardia spp.*	Immunomagnetic separation, nested-PCR and sequencing	18S rRNA gene	Raw and treated water samples	0.12-0.2 cysts and 0.04-0.1 oocysts per reaction	[120]
Giardia duodenalis	RFLP-PCR and gel electrophoresis	Triose phosphate isomerase gene	Wastewater samples	Data not available	[121]

Cryptosporidium spp.	Immunomagnetic separation, PCR and sequencing	18S rRNA gene and gp60 gene	Environmental water samples	Data not available	[122]
Cryptosporidium spp.	Immunomagnetic separation, RFLP-PCR, sequencing	18S rRNA gene and gp60 gene	Human fecal samples, drinking water, raw water and wastewater samples	Data not available	[123]
Giardia spp. and *Cryptosporidium spp.*	Semi-nested PCR, nested-PCR, nested real-time PCR TaqMan, gel electrophoresis and sequencing	*Giardia:* β-giardin gene (semi-nested PCR) and 18S rRNA gene (Nested real-time PCR)<br><br>*Cryptosporidium*: 18S rRNA gene (Nested PCR and nested real-time PCR)	Surface water samples	Data not available	[124]
Giardia spp. and *Cryptosporidium spp.*	Nested PCR, gel electrophoresis and sequencing	18S rRNA gene	Raw and treated water samples	10 oocysts/mL and 2 cysts/mL	[125]
Giardia spp. and *Cryptosporidium spp.*	Immunomagnetic separation and qPCR	*Giardia*: β-giardin gene<br><br>*Cryptosporidium*: COWP gene	Environmental water samples (spiked or not)	Data not available	[126]
Giardia duodenalis and *Cryptosporidium spp.*	Immunomagnetic separation, nested PCR, gel electrophoresis and sequencing	*Cryptosporidium*: 18S rRNA gene<br><br>*Giardia*: β-giardin gene	Raw and treated water samples	Data not available	[127]
Giardia duodenalis	RFLP-PCR, semi-nested PCR, gel electrophoresis and sequencing	β-giardin gene and glutamate dehydrogenase gene	Human and animal fecal samples, water samples, vegetable samples	Data not available	[128]
Giardia duodenalis and *Cryptosporidium spp.*	Immunomagnetic separation, PCR, nested PCR, gel electrophoresis and sequencing	*Giardia*: β-giardin gene, triose phosphate isomerase gene and	Human and animal fecal samples, water samples	Data not available	[129]

		glutamate dehydrogenase gene (PCR for all)<br><br>*Cryptosporidium*: 18S rRNA gene (nested PCR)			
Giardia spp. and *Cryptosporidium spp.*	Immunomagnetic separation, nested PCR, gel electrophoresis and sequencing	*Cryptosporidium*: 18S rRNA gene, HSP70 gene and gp60 gene<br><br>*Giardia*: β-giardin gene and triose phosphate isomerase gene	Surface water and treated water samples	Data not available	[130]
Giardia spp. and *Cryptosporidium spp.*	Immunomagnetic separation, PCR, nested PCR, gel electrophoresis and sequencing	*Cryptosporidium*: 18S rRNA gene and HSP70 gene (PCR)<br><br>*Giardia*: β-giardin gene and triose phosphate isomerase gene (nested PCR)	Recreational water samples	Data not available	[131]
Cryptosporidium spp.	Immunomagnetic separation, multiplex PCR, PCR, nested PCR and sequencing	18S rRNA gene and gp60 gene	Human fecal samples, river water samples and pool water samples	Data not available	[132]
Cryptosporidium parvum and *Giardia duodenalis*	Real-time PCR	18S rRNA gene	Purified (oo)cysts, raw water samples (spiked or not) and treated water samples (spiked or not)	Data not available	[133]
Cryptosporidium parvum and *Giardia duodenalis* Assemblage A	qPCR, multiplex qPCR, gel electrophoresis and sequencing	*Cryptosporidium*: COWP gene<br><br>*Giardia*: β-giardin gene	Wastewater samples and mussel samples	10 copies of target DNA per reaction	[134]

Cryptosporidium spp.	RFLP-Nested PCR and sequencing	18S rRNA gene and gp60 gene (sequencing only)	Human and animal fecal samples, environmental water samples	Data not available	[135]
Giardia sp.	Immunomagnetic separation and PCR	Glutamate dehydrogenase gene	Groundwater samples	Data not available	[136]
Giardia spp.	PCR and sequencing	18S rRNA gene	Human and animal fecal samples, water samples	Data not available	[137]
Cryptosporidium spp. and *Giardia* spp.	PCR, nested-PCR, gel electrophoresis and sequencing	18S rRNA gene	Environmental water samples	10 cells per reaction	[138]
Cryptosporidium spp. and *Giardia* spp.	Nested PCR, gel electrophoresis and sequencing	18S rRNA gene	Untreated and treated wastewater samples	Data not available	[139]
Cryptosporidium spp.	Immunomagnetic separation, nested PCR, gel electrophoresis, capillary electrophoresis and sequencing	18S rRNA gene<br><br>MM5, MM18, MM19, TP14, MS1 and MS9 loci	Animal fecal samples and environmental water samples	Data not available	[140]
Giardia spp.	Nested PCR, PCR, LAMP, gel electrophoresis and sequencing	18S rRNA gene (nested PCR) and glutamate dehydrogenase gene (PCR), αEF1 gene (LAMP)	Environmental water samples	100 fg of target DNA per mL of water	[141]
Cryptosporidium parvum and *Giardia lamblia*	Immunomagnetic separation and real-time PCR	*Cryptosporidium*: uncharacterized genomic sequence<br><br>*Giardia*: 18S rRNA gene	Environmental and treated water samples	Data not available	[142]
Cryptosporidium spp.	Immunomagnetic separation, replicate RFLP-nested PCR-and sequencing	18S rRNA gene	Environmental water samples	Data not available	[143]

Cryptosporidium spp.	Real-time PCR singleplex and duplex	18S rRNA gene	Treated water samples	Data not available	[144]
Cryptosporidium spp. and *Giardia spp.*	PCR, gel electrophoresis and sequencing	*Cryptosporidium*: gp60 gene<br><br>*Giardia*: Glutamate dehydrogenase gene	Purified (oo)cysts and water samples	6,5-65 fg of target DNA per µL (*Giardia*)<br><br>50 fg of target DNA per µL (*Cryptosporidium*)	[145]
Cryptosporidium spp. and *Giardia intestinalis*	PCR, nested PCR and sequencing	*Cryptosporidium*: 18S rRNA gene<br><br>*Giardia*: Glutamate dehydrogenase gene	Wastewater samples	Data not available	[146]
Cryptosporidium spp. and *Giardia intestinalis*	Multiplex real-time PCR	*Cryptosporidium*: DNA-J-like protein gene<br><br>*Giardia*: 18S rRNA gene	Wastewater samples	10 copies of target DNA per reaction	[147]
Giardia duodenalis	Nested PCR, gel electrophoresis and sequencing	β-giardin gene, triose phosphate isomerase gene and glutamate dehydrogenase gene	Wastewater samples	Data not available	[148]
Cryptosporidium spp. and *Giardia duodenalis*	Immunomagnetic separation, RFLP-nested PCR, nested PCR, gel electrophoresis and sequencing	*Cryptosporidium*: 18S rRNA gene (RFLP-nested PCR) and gp60 gene (nested PCR)<br><br>*Giardia*: glutamate dehydrogenase gene (RFLP-nested PCR)	River water samples	Data not available	[149]
Cryptosporidium spp	Immunomagnetic separation, RFLP-nested PCR, nested PCR, gel electrophoresis and sequencing	*Cryptosporidium*: 18S rRNA gene (RFLP-	Raw surface water samples	Data not available	[150]

		nested PCR) and gp60 gene (nested PCR)<br><br>*Giardia*: glutamate dehydrogenase gene			
Giardia intestinalis	Nested PCR, real-time PCR TaqMan, loop-mediated isothermal amplification, gel electrophoresis and sequencing	18S rRNA gene (nested PCR), β-giardin gene (real-time PCR) and EF1-α-gene (LAMP)	Environmental water samples	Data not available	[151]
Cryptosporidium spp. and *Giardia spp.*	Immunomagnetic separation, nested PCR, multiplex real-time PCR and sequencing	*Cryptosporidium*: 18S rRNA gene (nested PCR and multiplex real-time PCR), gp60 gene (nested PCR) and Lib13 locus (multiplex real-time PCR)<br><br>*Giardia*: β-giardin gene and triose phosphate isomerase gene (nested PCR)	Wastewater sample	Data not available	[152]
Cryptosporidium spp. and *Giardia spp.*	Immunomagnetic separation and nested PCR	*Cryptosporidium*: 18S rRNA gene<br><br>*Giardia*: β-giardin gene	Raw and treated water samples	Data not available	[153]
Cryptosporidium spp. and *Giardia spp.*	Nested PCR, cloning in a plasmidic vector and sequencing	18S rRNA gene	Swimming pool water samples	Data not available	[154]
Cryptosporidium spp. and *Giardia duodenalis*	Nested PCR, gel electrophoresis and sequencing	*Cryptosporidium*: 18S rRNA gene<br><br>*Giardia*: β-giardin gene and triose phosphate isomerase gene	Raw water samples	Data not available	[155]

Cryptosporidium parvum and *Giardia lamblia*	qPCR TaqMan	18S rRNA gene	Drinking water samples	1500 copies of target gene per litre of water	[156]
Cryptosporidium spp. and *Giardia intestinalis*	Immunomagnetic separation, nested PCR, real-time PCR TaqMan, cloning in a plasmidic vector and sequencing	*Cryptosporidium*: 18S rRNA gene<br><br>*Giardia*: glutamate dehydrogenase gene and 18S rRNA gene	Raw surface water samples	1–2 oocyst/L (Real-time PCR)	[157]
Cryptosporidium spp.	Nested PCR, gel electrophoresis ands sequencing	Gp60 gene	Pond and reservoir water samples	Data not available	[158]
Cryptosporidium spp. and *Giardia duodenalis*	Nested PCR and gel electrophoresis	*Cryptosporidium*: 18S rRNA gene<br><br>*Giardia*: Triose phosphate isomerase gene	Water and sludge samples	Data not available	[159]
Cryptosporidium spp. and *Giardia duodenalis*	Real-time PCR TaqMan, PCR, gel electrophoresis and sequencing	*Cryptosporidium*: 18S rRNA gene and gp60 gene<br><br>*Giardia*: 18S rRNA gene, glutamate dehydrogenase gene and triose phosphate isomerase gene	Raw and treated water samples	Data not available	[160]
Cryptosporidium spp. and *Giardia spp.*	Nested PCR and sequencing	18S rRNA gene	Recreational water samples	Data not available	[161] and [162]
Cryptosporidium spp.	qPCR, droplet digital PCR and Illumina MiSeq sequencing	18S rRNA gene and Clec gene	Wastewater samples	1 oocyst/µL of DNA extract	[163]

Cryptosporidium spp.	Immunomagnetic separation, nested PCR and sequencing	18S rRNA gene	Raw water samples	Data not available	[164]
Cryptosporidium spp. and *Giardia lamblia*	RFLP-nested PCR, gel electrophoresis and sequencing	*Cryptosporidium*: COWP gene<br><br>*Giardia*: β-giardin gene	Treated water samples	Data not available	[165]
Cryptosporidium spp. and *Giardia duodenalis*	Nested PCR, semi-nested PCR, gel electrophoresis and sequencing	18S rRNA gene	Environmental and sewage water samples	Data not available	[166]
Cryptosporidium spp. and *Giardia spp.*	Immunomagnetic separation, nested PCR and gel electrophoresis	*Cryptosporidium*: 18S rRNA gene<br><br>*Giardia*: Glutamate dehydrogenase gene and triose phosphate isomerase	Water samples	Data not available	[167]
Cryptosporidium spp.	Immunomagnetic separation, reverse transcriptase PCR, nested PCR, gel electrophoresis and sequencing	HSP70 gene (RT-PCR) and 18S rRNA gene (nested PCR)	Environmental and treated water samples	Data not available	[168]
Cryptosporidium spp. and *Giardia spp.*	Immunomagnetic separation, PCR, nested PCR and sequencing	*Cryptosporidium*: 18S rRNA gene<br><br>*Giardia*: 18S rRNA gene, glutamate dehydrogenase gene, triose phosphate isomerase gene and β-giardin gene	Treated water samples	Data not available	[169]
Giardia intestinalis	RFLP-Nested PCR, RFLP-heminested PCR, gel electrophoresis and DNA sequencing	Triose phosphate isomerase gene (RFLP-Nested PCR) and glutamate dehydrogenase	Human fecal samples, raw water samples and drinking water samples	Data not available	[170]

		gene (RFLP-heminested PCR)			
Giardia intestinalis	PCR and gel electrophoresis	18S rRNA gene	Human and bovine fecal samples, water samples	Data not available	[171]
Cryptosporidium spp. and *Giardia duodenalis*	PCR, gel electrophoresis and sequencing	*Cryptosporidium*: 18S rRNA gene and gp60 gene<br><br>*Giardia*: Triose phosphate isomerase gene and β-giardin gene	Wastewater and sludge samples	Data not available	[172]
Giardia lamblia	qPCR	18S rRNA gene	Environmental water samples	Data not available	[173]
Cryptosporidium spp. and *Giardia duodenalis*	(RFLP-) nested PCR, gel electrophoresis and sequencing	*Cryptosporidium*: 18S rRNA gene and gp60 gene<br><br>*Giardia*: glutamate dehydrogenase gene, triose phosphate isomerase gene and β-giardin gene	Wastewater samples	Data not available	[174]
Cryptosporidium spp. and *Giardia spp.*	Immunomagnetic separation, nested PCR, gel electrophoresis and sequencing	*Cryptosporidium*: 18S rRNA gene, gp60 gene and HSP70 gene<br><br>*Giardia*: 18S rRNA gene, β-giardin gene, glutamate dehydrogenase gene and triose phosphate isomerase	Mussel samples and wastewater samples	Data not available	[175]
Cryptosporidium spp. and *Giardia duodenalis*	Immunomagnetic separation, PCR, gel electrophoresis and sequencing	*Cryptosporidium*: 18S rRNA gene, HSP70 gene and gp60 gene	Environmental and treated water samples, animal fecal samples	Data not available	[176]

		Giardia: β-giardin gene, glutamate dehydrogenase gene and triose phosphate isomerase			
Cryptosporidium sp. and *Giardia sp*.	PCR and Illumina MiSeq sequencing	18S rRNA gene	Drinking water, reservoir water, groundwater, river water and one reclaimed water source samples	Data not available	[177]
Cryptosporidium spp. and *Giardia duodenalis*	qPCR	*Cryptosporidium*: 18S rRNA gene<br><br>*Giardia*: β-giardin gene	Groundwater samples	Data not available	[178]
Cryptosporidium sp.	Illumina MiSeq sequencing	18S rRNA gene	Raw, drinking and reservoir water samples	Data not available	[179]
Cryptosporidium spp. and *Giardia duodenalis*	Real-time PCR	*Cryptosporidium*: COWP gene<br><br>*Giardia*: 18S rRNA gene	Drinking water	Data not available	[180]
Cryptosporidium spp. and *Giardia spp*.	Droplet Digital PCR	*Cryptosporidium*: 18S rRNA gene<br><br>*Giardia*: β-giardin gene	Tap and reservoir water samples	Data not available	[181]
Cryptosporidium spp. and *Giardia spp*.	Nested PCR, gel electrophoresis and sequencing	*Cryptosporidium*: 18S rRNA gene<br><br>*Giardia*: triose phosphate isomerase gene	Surface water samples	0.4 (oo)cyst/L	[182]
Cryptosporidium sp.	Nested PCR, gel electrophoresis and sequencing	18S rRNA gene	Surface water samples	Data not available	[183]

Cryptosporidium spp.	Nested PCR, gel electrophoresis and sequencing	18S rRNA gene and gp60 gene	Surface, raw, treated and abattoir effluent water samples	Data not available	[184]
Cryptosporidium spp. and Giardia duodenalis	Nested PCR, gel electrophoresis and sequencing	*Cryptosporidium*: 18S rRNA gene and gp60 gene<br><br>*Giardia*: β-giardin gene, glutamate dehydrogenase gene and triose phosphate isomerase	Wastewater samples	Data not available	[185]
Giardia lamblia	Nanoscale qPCR	18S rRNA gene	Recreative water samples	Data not available	[186]
Cryptosporidium sp. and *Giardia* sp.	PCR	*Cryptosporidium*: 18S rRNA gene<br><br>*Giardia*: HSP70 gene	Raw, treated and backflushed water samples	0,01 parasites per 100 L of water	[187]
Giardia sp.	Real-time PCR	18S rRNA gene	Open drains, canals, floodwater, septic tanks, and anaerobic baffled reactors water samples	4 genome copies	[188]
Cryptosporidium spp.	qPCR, nested PCR, gel electrophoresis and Illumina MiSeq sequencing	18S rRNA gene (qPCR), gp60 gene (nested PCR and sequencing)	River water samples	Data not available	[189]
Cryptosporidium spp. and *Giardia duodenalis*	Nested Multiplex PCR, cloth-based hybridization array	*Giardia*: 16S rRNA gene and triose phosphate isomerase gene<br><br>*Cryptosporidium*: COWP gene	Wastewater and river water samples artificially contaminated	Data not available	[190]
Cryptosporidium parvum and *Giardia lamblia*	qPCR	*Giardia*: Glumate dehydrogenase gene and β-giardin gene	Artificially contaminated water samples	*Cryptosporidium*: 4 genome copies per PCR reaction	[191]

		Cryptosporidium: COWP gene and Lib13 gene		*Giardia*: 5 genome copies per PCR reaction	
Giardia duodenalis	(Semi-) nested PCR, gel electrophoresis and sequencing	18S rRNA gene (nested PCR) and glutamate dehydrogenase gene (semi-nested PCR)	Lake water samples	Data not available	[192]
Cryptosporidium sp.	Immunomagnetic separation, nested PCR and sequencing	18S rRNA gene and gp60 gene	River and lake water samples	Data not available	[193]

Note: For simplicity's sake in the table, complete titles such as the small-subunit gene and 18S rRNA gene were abbreviated to 18S rRNA gene in the table. Also, because most studies used Sanger technology, the heading ''sequencing'' used in this table refers to this technique. Next generation sequencing is specifically mentioned when it was used. It is important to explain that the limits of detection specified in this table are the ones clearly stated in the article itself. Any limit of detection present in supplementary data or mentioned in a previous article was not considered and classified as "Data not available" along with the other articles not presenting a limit of detection value.

References

[1] Abbaszadegan M, Gerba CP, Rose JB. Detection of *Giardia* cysts with a cDNA probe and applications to water samples. Appl Environ Microbiol 1991;57:927–31. https://doi.org/10.1128/aem.57.4.927-931.1991.

[2] Mahbubani MH, Bej AK, Perlin MH, Schaefer FW, Jakubowski W, Atlas RM. Differentiation of *Giardia duodenalis* from other *Giardia* spp. by using polymerase chain reaction and gene probes. J Clin Microbiol 1992;30:74–8. https://doi.org/10.1128/jcm.30.1.74-78.1992.

[3] Johnson DW, Pieniazek NJ, Rose JB. DNA probe hybridization and PCR detection of *Cryptosporidium* compared to immunofluorescence assay. Water Sci Technol 1993;27:77–84. https://doi.org/10.2166/wst.1993.0325.

[4] Johnson DW, Pieniazek NJ, Griffin DW, Misener L, Rose JB. Development of a PCR protocol for sensitive detection of *Cryptosporidium* oocysts in water samples. Appl Environ Microbiol 1995;61:3849–55. https://doi.org/10.1128/aem.61.11.3849-3855.1995.

[5] Laberge I, Ibrahim A, Barta JR, Griffiths MW. Detection of *Cryptosporidium parvum* in raw milk by PCR and oligonucleotide probe hybridization. Appl Environ Microbiol 1996;62:3259–64. https://doi.org/10.1128/aem.62.9.3259-3264.1996.

[6] Mayer CL, Palmer CJ. Evaluation of PCR, nested PCR, and fluorescent antibodies for detection of *Giardia* and *Cryptosporidium* species in wastewater. Appl Environ Microbiol 1996;62:2081–5.

[7] Stinear T, Matusan A, Hines K, Sandery M. Detection of a single viable *Cryptosporidium parvum* oocyst in environmental water concentrates by reverse transcription-PCR. Appl Environ Microbiol 1996;62:3385–90.

[8] Wallis PM, Erlandsen SL, Isaac-Renton JL, Olson ME, Robertson WJ, Van Keulen H. Prevalence of *Giardia* cysts and *Cryptosporidium* oocysts and characterization of *Giardia* spp isolated from drinking water in Canada. Appl Environ Microbiol 1996;62:2789–97. https://doi.org/10.1128/aem.62.8.2789-2797.1996.

[9] Rochelle PA, De Leon R, Stewart MH, Wolfe RL. Comparison of primers and optimization of PCR conditions for detection of *Cryptosporidium parvum* and *Giardia lamblia* in water. Appl Environ Microbiol 1997;63:106–14.

[10] Sluter SD, Tzipori S, Widmer G. Parameters affecting polymerase chain reaction detection of waterborne *Cryptosporidium parvum* oocysts. Appl Microbiol Biotechnol 1997;48:325–30.

[11] Chung E, Aldom JE, Chagla AH, Kostrzynska M, Lee H, Palmateer G, et al. Detection of *Cryptosporidium parvum* oocysts in municipal water samples by the polymerase chain reaction. J Microbiol Methods 1998;33:171–80. https://doi.org/10.1016/S0167-7012(98)00050-5.

[12] Deng MQ, Cliver DO. Differentiation of *Cryptosporidium parvum* isolates by a simplified randomly amplified polymorphic DNA technique. Appl Environ Microbiol 1998;64:1954–7.

[13] Gibbons CL, Rigi FM, Awad El-Kariem FM. Detection of *Cryptosporidium parvum* and *C. muris* Oocysts in Spiked Backwash Water using Three PCR-Based Protocols. Protist 1998;149:127–34. https://doi.org/10.1016/s1434-4610(98)70017-3.

[14] Kaucner C, Stinear T. Sensitive and rapid detection of viable *Giardia* cysts and *Cryptosporidium parvum* oocysts in large-volume water samples with wound fiberglass cartridge filters and reverse transcription-PCR. Appl Environ Microbiol 1998;64:1743–9.

[15] Chung E, Aldom JE, Carreno RA, Chagla AH, Kostrzynska M, Lee H, et al. PCR-based quantitation of *Cryptosporidium parvum* in municipal water samples. J Microbiol Methods 1999;38:119–30. https://doi.org/10.1016/S0167-7012(99)00087-1.
[16] Di Giovanni GD, Hashemi FH, Shaw NJ, Abrams FA, LeChevallier MW, Abbaszadegan M. Detection of infectious *Cryptosporidium parvum* oocysts in surface and filter backwash water samples by immunomagnetic separation and integrated cell culture-PCR. Appl Environ Microbiol 1999;65:3427–32.
[17] Hallier-Soulier S, Guillot E. An immunomagnetic separation polymerase chain reaction assay for rapid and ultra-sensitive detection of *Cryptosporidium parvum* in drinking water. FEMS Microbiol Lett 1999;176:285–9. https://doi.org/10.1016/S0378-1097(99)00246-3.
[18] Kostrzynska M, Sankey M, Haack E, Power C, Aldom JE, Chagla AH, et al. Three sample preparation protocols for polymerase chain reaction based detection of *Cryptosporidium parvum* in environmental samples. J Microbiol Methods 1999;35:65–71. https://doi.org/10.1016/S0167-7012(98)00106-7.
[19] Hallier-Soulier S, Guillot E. Detection of cryptosporidia and *Cryptosporidium parvum* oocysts in environmental water samples by immunomagnetic separation-polymerase chain reaction. J Appl Microbiol 2000;89:5–10. https://doi.org/10.1046/j.1365-2672.2000.01029.x.
[20] Lowery CJ, Moore JE, Millar BC, Burke DP, McCorry KAJ, Crothers E, et al. Detection and speciation of *Cryptosporidium* spp. in environmental water samples by immunomagnetic separation, PCR and endonuclease restriction. J Med Microbiol 2000;49:779–85. https://doi.org/10.1099/0022-1317-49-9-779.
[21] Mcintyre L, Hoang L, Ong CSL, Lee P, Isaac-Renton JL. Evaluation of Molecular Techniques to Biotype *Giardia duodenalis* Collected During an Outbreak. J Parasitol 2000;86:172–7.
[22] Udeh P, Veenstra J, Abraham AJ, John GH. Quantitative polymerase chain (QPCR) reaction using the MIMIC approach to estimate *Cryptosporidium parvum* oocysts, an intestinal pathogen, in municipal water treatment sludge samples. Mol Cell Probes 2000;14:121–6. https://doi.org/10.1006/mcpr.2000.0294.
[23] Xiao L, Alderisio K, Limor J, Royer M, Lal AA. Identification of species and sources of *Cryptosporidium* oocysts in storm waters with a small-subunit rRNA-based diagnostic and genotyping tool. Appl Environ Microbiol 2000;66:5492–8. https://doi.org/10.1128/AEM.66.12.5492-5498.2000.
[24] Lowery CJ, Moore JE, Millar BC, McCorry KAJ, Xu J, Rooney PJ, et al. Occurrence and molecular genotyping of *Cryptosporidium* spp. in surface waters in Northern Ireland. J Appl Microbiol 2001;91:774–9. https://doi.org/10.1046/j.1365-2672.2001.01440.x.
[25] Lowery CJ, Nugent P, Moore JE, Millar BC, Xiru X, Dooley JSG. PCR–IMS detection and molecular typing of *Cryptosporidium parvum* recovered from a recreational river source and an associated mussel (*Mytilus edulis*) bed in Northern Ireland. Epidemiol Infect 2001;127:545–53. https://doi.org/10.1017/s0950268801006276.
[26] Ono K, Tsuji H, Rai SK, Yamamoto A, Masuda K, Endo T, et al. Contamination of River Water by *Cryptosporidium parvum* Oocysts in Western Japan. Appl Environ Microbiol 2001;67:3832–6. https://doi.org/10.1128/AEM.67.9.3832-3836.2001.
[27] Rimhanen-Finne R, Ronkainen P, Hänninen ML. Simultaneous detection of *Cryptosporidium parvum* and *Giardia* in sewage

sludge by IC-PCR. J Appl Microbiol 2001;91:1030–5. https://doi.org/10.1046/j.1365-2672.2001.01468.x.
[28] Xiao L, Singh A, Limor J, Graczyk TK, Gradus S, Lal AA. Molecular Characterization of *Cryptosporidium* Oocysts in Samples of Raw Surface Water and Wastewater. Appl Environ Microbiol 2001;67:1097–101. https://doi.org/10.1128/AEM.67.3.1097-1101.2001.
[29] Heitman TL, Frederick LM, Viste JR, Guselle NJ, Morgan UM, Thompson RCA, et al. Prevalence of *Giardia* and *Cryptosporidium* and characterization of *Cryptosporidium* spp. isolated from wildlife, human, and agricultural sources in the North Saskatchewan River Basin in Alberta, Canada. Can J Microbiol 2002;48:530–41. https://doi.org/10.1139/w02-047.
[30] Jellison KL, Hemond HF, Schauer DB. Sources and species of *Cryptosporidium* oocysts in the Wachusett Reservoir watershed. Appl Environ Microbiol 2002;68:569–75. https://doi.org/10.1128/AEM.68.2.569-575.2002.
[31] Rimhanen-Finne R, Hörman A, Ronkainen P, Hänninen ML. An IC-PCR method for detection of *Cryptosporidium* and *Giardia* in natural surface waters in Finland. J Microbiol Methods 2002;50:299–303. https://doi.org/10.1016/S0167-7012(02)00047-7.
[32] Sturbaum GD, Klonicki PT, Marshall MM, Jost BH, Clay BL, Sterling CR. Immunomagnetic separation (IMS)-fluorescent antibody detection and IMS-PCR detection of seeded *Cryptosporidium parvum* oocysts in natural waters and their limitations. Appl Environ Microbiol 2002;68:2991–6. https://doi.org/10.1128/AEM.68.6.2991-2996.2002.
[33] Van Keulen H, Macechko PT, Wade S, Schaaf S, Wallis PM, Erlandsen SL. Presence of human *Giardia* in domestic, farm and wild animals, and environmental samples suggests a zoonotic potential for *Giardia*sis. Vet Parasitol 2002;108:97–107. https://doi.org/10.1016/S0304-4017(02)00181-4.
[34] Ward PI, Deplazes P, Regli W, Rinder H, Mathis A. Detection of eight *Cryptosporidium* genotypes in surface and waste waters in Europe. Parasitology 2002;124:359–68. https://doi.org/10.1017/S0031182001001317.
[35] Cacciò SM, De Giacomo M, Aulicino FA, Pozio E. *Giardia* cysts in wastewater treatment plants in Italy. Appl Environ Microbiol 2003;69:3393–8. https://doi.org/10.1128/AEM.69.6.3393-3398.2003.
[36] Hallier-Soulier S, Guillot E. An immunomagnetic separation-reverse transcription polymerase chain reaction (IMS-RT-PCR) test for sensitive and rapid detection of viable waterborne *Cryptosporidium parvum*. Environ Microbiol 2003;5:592–8.
[37] Higgins JA, Trout JM, Fayer R, Shelton DR, Jenkins MC. Recovery and detection of *Cryptosporidium parvum* oocysts from water samples using continuous flow centrifugation. Water Res 2003;37:3551–60. https://doi.org/10.1016/S0043-1354(03)00251-3.
[38] Lechevallier MW, Di Giovanni GD, Bukhari Z, Bukhari S, Rosen JS, Sobrinho J, et al. Comparison of Method 1623 and Cell Culture-PCR for Detection of *Cryptosporidium* spp. in Source Waters. Appl Environ Microbiol 2003;69:971–9. https://doi.org/10.1128/AEM.69.2.971.
[39] Quintero-Betancourt W, Gennaccaro AL, Scott TM, Rose JB. Assessment of methods for detection of infectious *Cryptosporidium* oocysts and *Giardia* cysts in reclaimed effluents. Appl Environ Microbiol 2003;69:5380–8. https://doi.org/10.1128/AEM.69.9.5380-5388.2003.
[40] Tsuchihashi R, Loge FJ, Darby JL. Detection of *Cryptosporidium parvum* in Secondary Effluents Using a Most Probable Number-Polymerase Chain Reaction Assay. Water Environ Res 2003;75:292–9. https://doi.org/10.2175/106143003x141097.

[41] Ali MA, Al-Herrawy AZ, El-Hawaary SE. Detection of enteric viruses, *Giardia* and *Cryptosporidium* in two different types of drinking water treatment facilities. Water Res 2004;38:3931–9. https://doi.org/10.1016/j.watres.2004.06.014.
[42] Bertrand I, Gantzer C, Chesnot T, Schwartzbrod J. Improved specificity for *Giardia lamblia* cyst quantification in wastewater by development of a real-time PCR method. J Microbiol Methods 2004;57:41–53. https://doi.org/10.1016/j.mimet.2003.11.016.
[43] Horman A, Rimhanen-Finne R, Maunula L, von Bonsdorff CH, Torvela N, Heikinheimo A, et al. *Campylobacter* spp., *Giardia* spp., *Cryptosporidium* spp., Noroviruses, and Indicator Organisms in Surface Water in Southwestern Finland, 2000-2001. Appl Environ Microbiol 2004;70:87–95. https://doi.org/10.1128/AEM.70.1.87.
[44] Sulaiman IM, Jiang J, Singh A, Xiao L. Distribution of *Giardia duodenalis* genotypes and subgenotypes in raw urban wastewater in Milwaukee, Wisconsin. Appl Environ Microbiol 2004;70:3776–80. https://doi.org/10.1128/AEM.70.6.3776-3780.2004.
[45] Hänninen ML, Hörman A, Rimhanen-Finne R, Vahtera H, Malmberg S, Herve S, et al. Monitoring of *Cryptosporidium* and *Giardia* in the Vantaa river basin, southern Finland. Int J Hyg Environ Health 2005;208:163–71. https://doi.org/10.1016/j.ijheh.2005.01.026.
[46] Jiang J, Alderisio KA, Singh A, Xiao L. Development of Procedures for Direct Extraction of *Cryptosporidium* DNA from Water Concentrates and for Relief of PCR Inhibitors. Appl Environ Microbiol 2005;71:1135–41. https://doi.org/10.1128/AEM.71.3.1135.
[47] Ryan UM, Read CM, Hawkins P, Warnecke M, Swanson P, Griffith M, et al. Genotypes of *Cryptosporidium* from Sydney water catchment areas. J Appl Microbiol 2005;98:1221–9. https://doi.org/10.1111/j.1365-2672.2005.02562.x.
[48] Carey CM, Lee H, Trevors JT. Comparison of most probable number-PCR and most probable number-foci detection method for quantifying infectious *Cryptosporidium parvum* oocysts in environmental samples. J Microbiol Methods 2006;67:363–72. https://doi.org/10.1016/j.mimet.2006.04.007.
[49] Hashimoto A, Sugimoto H, Morita S, Hirata T. Genotyping of single *Cryptosporidium* oocysts in sewage by semi-nested PCR and direct sequencing. Water Res 2006;40:2527–32. https://doi.org/10.1016/j.watres.2006.04.038.
[50] Hirata T, Hashimoto A. Genotyping of single *Cryptosporidium* oocysts isolated from sewage and river water. Water Sci Technol 2006;54:197–202. https://doi.org/10.2166/wst.2006.469.
[51] Lonigro A, Pollice A, Spinelli R, Berrilli F, Di Cave D, D'Orazi C, et al. *Giardia* cysts and *Cryptosporidium* oocysts in membrane-filtered municipal wastewater used for irrigation. Appl Environ Microbiol 2006;72:7916–8. https://doi.org/10.1128/AEM.01903-06.
[52] Masago Y, Oguma K, Katayama H, Ohgaki S. Quantification and genotyping of *Cryptosporidium* spp. in river water by quenching probe PCR and denaturing gradient gel electrophoresis. Water Sci Technol 2006;54:119–26. https://doi.org/10.2166/wst.2006.457.
[53] Robertson LJ, Hermansen L, Gjerde BK. Occurrence of *Cryptosporidium* oocysts and *Giardia* cysts in sewage in Norway. Appl Environ Microbiol 2006;72:5297–303. https://doi.org/10.1128/AEM.00464-06.
[54] Robertson LJ, Hermansen L, Gjerde BK, Strand E, Alvsvåg JO, Langeland N. Application of genotyping during an extensive

outbreak of waterborne *Giardia*sis in Bergen, Norway, during autumn and winter 2004. Appl Environ Microbiol 2006;72:2212–7. https://doi.org/10.1128/AEM.72.3.2212-2217.2006.
[55] Xiao L, Alderisio KA, Jiang J. Detection of *Cryptosporidium* oocysts in water: Effect of the number of samples and analytic replicates on test results. Appl Environ Microbiol 2006;72:5942–7. https://doi.org/10.1128/AEM.00927-06.
[56] Anceno AJ, Katayama H, Houpt ER, Chavalitshewinkoon-Petmitr P, Chuluun B, Shipin O V. IMS-free DNA extraction for the PCR-based quantification of *Cryptosporidium parvum* and *Giardia lamblia* in surface and waste water. Int J Environ Health Res 2007;17:297–310. https://doi.org/10.1080/09603120701372573.
[57] Bertrand I, Schwartzbrod J. Detection and genotyping of *Giardia duodenalis* in wastewater: Relation between assemblages and faecal contamination origin. Water Res 2007;41:3675–82. https://doi.org/10.1016/j.watres.2007.02.043.
[58] Ahmed W, Huygens F, Goonetilleke A, Gardner T. Real-time PCR detection of pathogenic microorganisms in roof-harvested rainwater in Southeast Queensland, Australia. Appl Environ Microbiol 2008;74:5490–6. https://doi.org/10.1128/AEM.00331-08.
[59] Castro-Hermida JA, García-Presedo I, Almeida A, González-Warleta M, Correia Da Costa JM, Mezo M. Presence of *Cryptosporidium* spp. and *Giardia duodenalis* through drinking water. Sci Total Environ 2008;405:45–53. https://doi.org/10.1016/j.scitotenv.2008.06.040.
[60] Keeley A, Faulkner BR. Influence of land use and watershed characteristics on protozoa contamination in a potential drinking water resources reservoir. Water Res 2008;42:2803–13. https://doi.org/10.1016/j.watres.2008.02.028.
[61] Plutzer J, Karanis P, Domokos K, Törökné A, Márialigeti K. Detection and characterisation of *Giardia* and *Cryptosporidium* in Hungarian raw, surface and sewage water samples by IFT, PCR and sequence analysis of the SSUrRNA and GDH genes. Int J Hyg Environ Health 2008;211:524–33. https://doi.org/10.1016/j.ijheh.2008.04.004.
[62] Yang W, Chen P, Villegas EN, Landy RB, Kanetsky C, Cama VA, et al. *Cryptosporidium* source tracking in the potomac river watershed. Appl Environ Microbiol 2008;74:6495–504. https://doi.org/10.1128/AEM.01345-08.
[63] Castro-Hermida JA, García-Presedo I, Almeida A, González-Warleta M, Correia Da Costa JM, Mezo M. Detection of *Cryptosporidium* spp. and *Giardia duodenalis* in surface water: A health risk for humans and animals. Water Res 2009;43:4133–42. https://doi.org/10.1016/j.watres.2009.06.020.
[64] Feng Y, Li N, Duan L, Xiao L. *Cryptosporidium* genotype and subtype distribution in raw wastewater in Shanghai, China: Evidence for possible unique *Cryptosporidium hominis* transmission. J Clin Microbiol 2009;47:153–7. https://doi.org/10.1128/JCM.01777-08.
[65] Inomata A, Kishida N, Momoda T, Akiba M, Izumiyama S, Yagita K, et al. Development and evaluation of a reverse transcription-loop-mediated isothermal amplification assay for rapid and high-sensitive detection of *Cryptosporidium* in water samples. Water Sci Technol 2009;60:2167–72. https://doi.org/10.2166/wst.2009.599.
[66] Lobo ML, Xiao L, Antunes F, Matos O. Occurrence of *Cryptosporidium* and *Giardia* genotypes and subtypes in raw and treated water in Portugal. Lett Appl Microbiol 2009;48:732–7. https://doi.org/10.1111/j.1472-765X.2009.02605.x.
[67] Alonso JL, Amorós I, Cuesta G. LNA probes in a real-time TaqMan PCR assay for genotyping of *Giardia duodenalis* in

wastewaters. J Appl Microbiol 2010;108:1594–601. https://doi.org/10.1111/j.1365-2672.2009.04559.x.
[68] Almeida A, Moreira MJ, Soares S, de Lurdes Delgado M, Figueiredo J, Silva E, et al. Biological and genetic characterization of *Cryptosporidium* spp. and *Giardia duodenalis* isolates from five hydrographical basins in northern Portugal. Korean J Parasitol 2010;48:105–11. https://doi.org/10.3347/kjp.2010.48.2.105.
[69] Almeida A, Moreira MJ, Soares S, De Delgado ML, Figueiredo J, Silva E, et al. Presence of *Cryptosporidium* spp. and *Giardia duodenalis* in drinking water samples in the North of Portugal. Korean J Parasitol 2010;48:43–8. https://doi.org/10.3347/kjp.2010.48.1.43.
[70] Betancourt WQ, Querales L, Sulbaran YF, Rodriguez-Diaz J, Caraballo L, Pujol FH. Molecular characterization of sewage-borne pathogens and detection of sewage markers in an urban stream in Caracas, Venezuela. Appl Environ Microbiol 2010;76:2023–6. https://doi.org/10.1128/AEM.02752-09.
[71] Dungeni M, Momba MNB. The abundance of *Cryptosporidium* and *Giardia* spp. in treated effluents produced by four wastewater treatment plants in the Gauteng Province of South Africa. Water SA 2010;36:425–32.
[72] Jenkins MB, Liotta JL, Lucio-Forster A, Bowman DD. Concentrations, viability, and distribution of *Cryptosporidium* genotypes in lagoons of swine facilities in the southern Piedmont and in coastal plain watersheds of Georgia. Appl Environ Microbiol 2010;76:5757–63. https://doi.org/10.1128/AEM.00434-10.
[73] Khouja LBA, Cama VA, Xiao L. Parasitic contamination in wastewater and sludge samples in Tunisia using three different detection techniques. Parasitol Res 2010;107:109–16. https://doi.org/10.1007/s00436-010-1844-8.
[74] Lee DY, Seto P, Korczak R. DNA microarray-based detection and identification of waterborne protozoan pathogens. J Microbiol Methods 2010;80:129–33. https://doi.org/10.1016/j.mimet.2009.11.015.
[75] Naeini KM, Asadi M, Chaleshtori MH. Detection and Molecular Characterization of *Cryptosporidium* species in Recreational Waters of Chaharmahal va Bakhtiyari Province of Iran using nested-PCR-RFLP. Iran J Parasitol 2010;6:20–7.
[76] Nichols RAB, Connelly L, Sullivan CB, Smith H V. Identification of *Cryptosporidium* species and genotypes in Scottish raw and drinking waters during a one-year monitoring period. Appl Environ Microbiol 2010;76:5977–86. https://doi.org/10.1128/AEM.00915-10.
[77] Santos SFO, Silva HD, Souza Júnior ES, Anunciação CE, Silveira-Lacerda EP, Vilanova-Costa CAST, et al. Environmental Monitoring of Opportunistic Protozoa in Rivers and Lakes in the Neotropics Based on Yearly Monitoring. Water Qual Expo Heal 2010;2:97–104. https://doi.org/10.1007/s12403-010-0027-2.
[78] Alonso JL, Amorós I, Cañigral I. Development and evaluation of a real-time PCR assay for quantification of *Giardia* and *Cryptosporidium* in sewage samples. Appl Microbiol Biotechnol 2011;89:1203–11. https://doi.org/10.1007/s00253-010-2984-6.
[79] Araújo RS, Dropa M, Fernandes LN, Carvalho TT, Sato MIZ, Soares RM, et al. Genotypic characterization of *Cryptosporidium hominis* from water samples in São Paulo, Brazil. Am J Trop Med Hyg 2011;85:834–8. https://doi.org/10.4269/ajtmh.2011.10-0449.
[80] Feng YY, Zhao X, Chen J, Jin W, Zhou X, Li N, et al. Occurrence, source, and human infection potential of *Cryptosporidium* and *Giardia* spp. in source and tap water in Shanghai, China. Appl Environ Microbiol 2011;77:3609–16.

https://doi.org/10.1128/AEM.00146-11.
[81] Fernandes LN, De Souza PP, De Araújo RS, Razzolini MTP, Soares RM, Sato MIZ, et al. Detection of assemblages A and B of *Giardia duodenalis* in water and sewage from São Paulo state, Brazil. J Water Health 2011;9:361–7. https://doi.org/10.2166/wh.2011.098.
[82] Helmi K, Skraber S, Burnet JB, Leblanc L, Hoffmann L, Cauchie HM. Two-year monitoring of *Cryptosporidium parvum* and *Giardia lamblia* occurrence in a recreational and drinking water reservoir using standard microscopic and molecular biology techniques. Environ Monit Assess 2011;179:163–75. https://doi.org/10.1007/s10661-010-1726-7.
[83] Koloren Z, Sotiriadou I, Karanis P. Investigations and comparative detection of *Cryptosporidium* species by microscopy, nested PCR and LAMP in water supplies of Ordu, Middle Black Sea, Turkey. Ann Trop Med Parasitol 2011;105:607–15. https://doi.org/10.1179/2047773211Y.0000000011.
[84] Liu A, Ji H, Wang E, Liu J, Xiao L, Shen Y, et al. Molecular identification and distribution of *Cryptosporidium* and *Giardia duodenalis* in raw urban wastewater in Harbin, China. Parasitol Res 2011;109:913–8. https://doi.org/10.1007/s00436-011-2333-4.
[85] Mahmoudi MR, Ashrafi K, Abedinzadeh H, Tahvildar-Bideruni F, Haghighi A, Bandehpour M, et al. Development of Sensitive Detection of *Cryptosporidium* and *Giardia* from Surface Water in Iran. Iran J Parasitol 2011;6:43–51.
[86] Ajonina C, Buzie C, Ajonina IU, Basner A, Reinhardt H, Gulyas H, et al. Occurrence of *Cryptosporidium* in a wastewater treatment plant in north Germany. J Toxicol Environ Heal - Part A Curr Issues 2012;75:1351–8. https://doi.org/10.1080/15287394.2012.721167.
[87] Ayed LB, Yang W, Widmer G, Cama VA, Ortega Y, Xiao L. Survey and genetic characterization of wastewater in Tunisia for *Cryptosporidium* spp., *Giardia duodenalis*, *Enterocytozoon bieneusi*, *Cyclospora cayetanensis* and *Eimeria* spp. J Water Health 2012;10:431–44. https://doi.org/10.2166/wh.2012.204.
[88] Budu-Amoako E, Greenwood SJ, Dixon BR, Barkema HW, McClure JT. Occurrence of *Cryptosporidium* and *Giardia* on beef farms and water sources within the vicinity of the farms on Prince Edward Island, Canada. Vet Parasitol 2012;184:1–9. https://doi.org/10.1016/j.vetpar.2011.10.027.
[89] Dungan RS, Klein M, Leytem AB. Quantification of bacterial indicators and zoonotic pathogens in dairy wastewater ponds. Appl Environ Microbiol 2012;78:8089–95. https://doi.org/10.1128/AEM.02470-12.
[90] Füchslin HP, Koïzsch S, Egli T. *Cryptosporidium* spp. in drinking water: Samples from rural sites in Switzerland. Swiss Med Wkly 2012;142:1–8. https://doi.org/10.4414/smw.2012.13683.
[91] Haramoto E, Katayama H, Asami M, Akiba M. Development of a novel method for simultaneous concentration of viruses and protozoa from a single water sample. J Virol Methods 2012;182:62–9. https://doi.org/10.1016/j.jviromet.2012.03.011.
[92] Keserue HA, Füchslin HP, Wittwer M, Nguyen-Viet H, Nguyen TT, Surinkul N, et al. Comparison of rapid methods for detection of *Giardia* spp. and *Cryptosporidium* spp. (Oo)cysts using transportable instrumentation in a field deployment. Environ Sci Technol 2012;46:8952–9. https://doi.org/10.1021/es301974m.
[93] Kishida N, Miyata R, Furuta A, Izumiyama S, Tsuneda S, Sekiguchi Y, et al. Quantitative detection of *Cryptosporidium* oocyst

in water source based on 18S rRNA by alternately binding probe competitive reverse transcription polymerase chain reaction (ABC-RT-PCR). Water Res 2012;46:187–94. https://doi.org/10.1016/j.watres.2011.10.048.
[94] Li N, Xiao L, Wang L, Zhao S, Zhao X, Duan L, et al. Molecular Surveillance of *Cryptosporidium* spp., *Giardia duodenalis*, and *Enterocytozoon bieneusi* by Genotyping and Subtyping Parasites in Wastewater. PLoS Negl Trop Dis 2012;6:1–12. https://doi.org/10.1371/journal.pntd.0001809.
[95] Liang Z, Keeley A. Comparison of propidium monoazide-quantitative PCR and reverse transcription quantitative PCR for viability detection of fresh *Cryptosporidium* oocysts following disinfection and after long-term storage in water samples. Water Res 2012;46:5941–53. https://doi.org/10.1016/j.watres.2012.08.014.
[96] Loganthan S, Yang R, Bath A, Gordon C, Ryan UM. Prevalence of *Cryptosporidium* species in recreational versus non-recreational water sources. Exp Parasitol 2012;131:399–403. https://doi.org/10.1016/j.exppara.2012.04.015.
[97] Rodríguez DC, Pino N, Peñuela G. Microbiological quality indicators in waters of dairy farms: Detection of pathogens by PCR in real time. Sci Total Environ 2012;427–428:314–8. https://doi.org/10.1016/j.scitotenv.2012.03.052.
[98] Ruecker NJ, Matsune JC, Wilkes G, Lapen DR, Topp E, Edge TA, et al. Molecular and phylogenetic approaches for assessing sources of *Cryptosporidium* contamination in water. Water Res 2012;46:5135–50. https://doi.org/10.1016/j.watres.2012.06.045.
[99] Van Dyke MI, Ong CSL, Prystajecky NA, Isaac-Renton JL, Huck PM. Identifying host sources, humanhealth risk and indicators of *Cryptosporidium* and *Giardia* in a Canadian watershed influenced by urban and rural activities. J Water Health 2012;10:311–23. https://doi.org/10.2166/wh.2012.131.
[100] Xiao S, An W, Chen Z, Zhang D, Yu J, Yang M. Occurrences and genotypes of *Cryptosporidium* oocysts in river network of southern-eastern China. Parasitol Res 2012;110:1701–9. https://doi.org/10.1007/s00436-011-2688-6.
[101] Damiani C, Balthazard-Accou K, Clervil E, Diallo A, Da Costa C, Emmanuel E, et al. Cryptosporidiosis in Haiti: Surprisingly low level of species diversity revealed by molecular characterization of *Cryptosporidium* oocysts from surface water and groundwater. Parasite 2013;20:1–6. https://doi.org/10.1051/parasite/2013045.
[102] Edge TA, Khan IUH, Bouchard R, Guo J, Hill S, Locas A, et al. Occurrence of waterborne pathogens and *Escherichia coli* at offshore drinking water intakes in lake Ontario. Appl Environ Microbiol 2013;79:5799–813. https://doi.org/10.1128/AEM.00870-13.
[103] Mahmoudi MR, Kazemi B, Mohammadiha A, Mirzaei A, Karanis P. Detection of *Cryptosporidium* and *Giardia* (oo) cysts by IFA , PCR and LAMP in surface water from Rasht, Iran. Trans R Soc Trop Med Hyg 2013;107:511–7. https://doi.org/10.1093/trstmh/trt042.
[104] Osaki SC, Soccol VT, Costa AO, Oliveira-Silva MB, Pereira JT, Procópio AE. Polymerase chain reaction and nested-PCR approaches for detecting *Cryptosporidium* in water catchments of water treatment plants in Curitiba, State of Paraná, Brazil. Rev Soc Bras Med Trop 2013;46:270–6. https://doi.org/10.1590/0037-8682-0053-2013.
[105] Ruecker NJ, Matsune JC, Lapen DR, Topp E, Edge TA, Neumann NF. The detection of *Cryptosporidium* and the resolution of mixtures of species and genotypes from water. Infect Genet Evol 2013;15:3–9. https://doi.org/10.1016/j.meegid.2012.09.009.
[106] Sroka J, Stojecki K, Zdybel J, Karamon J, Cencek T, Dutkiewicz J. Occurrence of *Cryptosporidium* oocysts and *Giardia* cysts

in effluent from sewage treatment plant from eastern Poland. Ann Agric Environ Med 2013;. 1:57–62.
[107] Staggs SE, Beckman EM, Keely SP, Mackwan R, Ware MW, Moyer AP, et al. The Applicability of TaqMan-Based Quantitative Real-Time PCR Assays for Detecting and Enumerating *Cryptosporidium* spp. Oocysts in the Environment. PLoS One 2013;8. https://doi.org/10.1371/journal.pone.0066562.
[108] Wilkes G, Ruecker NJ, Neumann NF, Gannon VPJ, Jokinen C, Sunohara M, et al. Spatiotemporal analysis of *Cryptosporidium* species/genotypes and relationships with other zoonotic pathogens in surface water from mixed-use watersheds. Appl Environ Microbiol 2013;79:434–48. https://doi.org/10.1128/AEM.01924-12.
[109] Xiao G, Qiu Z, Qi J, Chen JA, Liu F, Liu W, et al. Occurrence and potential health risk of *Cryptosporidium* and *Giardia* in the Three Gorges Reservoir, China. Water Res 2013;47:2431–45. https://doi.org/10.1016/j.watres.2013.02.019.
[110] Alonso JL, Amorós I, Guy RA. Quantification of viable *Giardia* cysts and *Cryptosporidium* oocysts in wastewater using propidium monoazide quantitative real-time PCR. Parasitol Res 2014;113:2671–8. https://doi.org/10.1007/s00436-014-3922-9.
[111] Betancourt WQ, Duarte DC, Vásquez RC, Gurian PL. *Cryptosporidium* and *Giardia* in tropical recreational marine waters contaminated with domestic sewage: Estimation of bathing-associated disease risks. Mar Pollut Bull 2014;85:268–73. https://doi.org/10.1016/j.marpolbul.2014.05.059.
[112] Dobrowsky PH, De Kwaadsteniet M, Cloete TE, Khan W. Distribution of indigenous bacterial pathogens and potential pathogens associated with roof-harvested rainwater. Appl Environ Microbiol 2014;80:2307–16. https://doi.org/10.1128/AEM.04130-13.
[113] Dreelin EA, Ives RL, Molloy S, Rose JB. *Cryptosporidium* and *Giardia* in surface water: A case study from Michigan, USA to inform management of rural water systems. Int J Environ Res Public Health 2014;11:10480–503. https://doi.org/10.3390/ijerph111010480.
[114] Durigan M, Abreu AG, Zucchi MI, Franco RMB, De Souza AP. Genetic diversity of *Giardia duodenalis*: Multilocus genotyping reveals zoonotic potential between clinical and environmental sources in a metropolitan region of Brazil. PLoS One 2014;9:1–28. https://doi.org/10.1371/journal.pone.0115489.
[115] Galván AL, Magnet A, Izquierdo F, Fernández Vadillo C, Peralta RH, Angulo S, et al. A year-long study of *Cryptosporidium* species and subtypes in recreational, drinking and wastewater from the central area of Spain. Sci Total Environ 2014;468–469:368–75. https://doi.org/10.1016/j.scitotenv.2013.08.053.
[116] Hu Y, Feng YY, Huang C, Xiao L. Occurrence, source, and human infection potential of *Cryptosporidium* and *Enterocytozoon bieneusi* in drinking source water in Shanghai, China, during a pig carcass disposal incident. Environ Sci Technol 2014;48:14219–27. https://doi.org/10.1021/es504464t.
[117] Kitajima M, Haramoto E, Iker BC, Gerba CP. Occurrence of *Cryptosporidium*, *Giardia*, and *Cyclospora* in influent and effluent water at wastewater treatment plants in Arizona. Sci Total Environ 2014;484:129–36. https://doi.org/10.1016/j.scitotenv.2014.03.036.
[118] Ma L, Sotiriadou I, Cai Q, Karanis G, Wang G, Wang G, et al. Detection of *Cryptosporidium* and *Giardia* in agricultural and water environments in the Qinghai area of China by IFT and PCR. Parasitol Res 2014;113:3177–84.

https://doi.org/10.1007/s00436-014-3979-5.
[119] Moss JA, Gordy J, Snyder RA. Effective Concentration and Detection of *Cryptosporidium*, *Giardia*, and the *Microsporidia* from Environmental Matrices. J Pathog 2014;2014:1–10. https://doi.org/10.1155/2014/408204.
[120] Prystajecky NA, Huck PM, Schreier H, Isaac-Renton JL. Assessment of *Giardia* and *Cryptosporidium* spp. as a microbial source tracking tool for surface water: Application in a mixed-use watershed. Appl Environ Microbiol 2014;80:2328–36. https://doi.org/10.1128/AEM.02037-13.
[121] Samie A, Ntekele P. Genotypic detection and evaluation of the removal efficiency of *Giardia duodenalis* at municipal wastewater treatment plants in Northern South Africa. Trop Biomed 2014;31:122–33.
[122] Swaffer BA, Vial HM, King BJ, Daly R, Frizenschaf J, Monis PT. Investigating source water *Cryptosporidium* concentration, species and infectivity rates during rainfall-runoff in a multi-use catchment. Water Res 2014;67:310–20. https://doi.org/10.1016/j.watres.2014.08.055.
[123] Widerström M, Schönning C, Lilja M, Lebbad M, Ljung T, Allestam G, et al. Large outbreak of *Cryptosporidium hominis* infection transmitted through the public water supply, Sweden. Emerg Infect Dis 2014;20:581–9. https://doi.org/10.3201/eid2004.121415.
[124] Adamska M, Sawczuk M, Kolodziejczyk L, Skotarczak B. Assessment of molecular methods as a tool for detecting pathogenic protozoa isolated from water bodies. J Water Health 2015;13:953–9. https://doi.org/10.2166/wh.2015.077.
[125] Almeida JC, Martins FDC, Ferreira Neto JM, dos Santos MM, Garcia JL, Navarro IT, et al. Occurrence of *Cryptosporidium* spp. and *Giardia* spp. in a public water-treatment system, Paraná, Southern Brazil. Rev Bras Parasitol Veterinária 2015;24:303–8. https://doi.org/10.1590/s1984-29612015051.
[126] Alfredo Bonilla J, Bonilla TD, Abdelzaher A, Scott TM, Lukasik JO, Solo-Gabriele HM, et al. Quantification of protozoa and viruses from small water volumes. Int J Environ Res Public Health 2015;12:7118–32. https://doi.org/10.3390/ijerph120707118.
[127] Castro-Hermida JA, González-Warleta M, Mezo M. *Cryptosporidium* spp. and *Giardia duodenalis* as pathogenic contaminants of water in Galicia, Spain: The need for safe drinking water. Int J Hyg Environ Health 2015;218:132–8. https://doi.org/10.1016/j.ijheh.2014.09.001.
[128] Colli CM, Bezagio RC, Nishi L, Bignotto TS, Ferreira ÉC, Falavigna-Guilherme AL, et al. Identical assemblage of *Giardia duodenalis* in humans, animals and vegetables in an urban area in Southern Brazil indicates a relationship among them. PLoS One 2015;10:1–12. https://doi.org/10.1371/journal.pone.0118065.
[129] David EB, Guimarães S, De Oliveira AP, De Oliveira-Sequeira TCG, Bittencourt GN, Nardi ARM, et al. Molecular characterization of intestinal protozoa in two poor communities in the State of São Paulo, Brazil. Parasites and Vectors 2015;8:1–12. https://doi.org/10.1186/s13071-015-0714-8.
[130] Ehsan A, Geurden T, Casaert S, Paulussen J, De Coster L, Schoemaker T, et al. Occurrence and potential health risk of *Cryptosporidium* and *Giardia* in different water catchments in Belgium. Environ Monit Assess 2015;187. https://doi.org/10.1007/s10661-014-4157-z.
[131] Ehsan MA, Casaert S, Levecke B, Van Rooy L, Pelicaen J, Smis A, et al. *Cryptosporidium* and *Giardia* in recreational water in

Belgium. J Water Health 2015;13:870–8. https://doi.org/10.2166/wh.2015.268.
[132] Gertler M, Dürr M, Renner P, Poppert S, Askar M, Breidenbach J, et al. Outbreak of following river flooding in the city of Halle (Saale), Germany, August 2013. BMC Infect Dis 2015;15:1–10. https://doi.org/10.1186/s12879-015-0807-1.
[133] Hill VR, Narayanan J, Gallen RR, Ferdinand KL, Cromeans TL, Vinjé J. Development of a nucleic acid extraction procedure for simultaneous recovery of DNA and RNA from diverse microbes in water. Pathogens 2015;4:335–54. https://doi.org/10.3390/pathogens4020335.
[134] Marangi M, Giangaspero A, Lacasella V, Lonigro A, Gasser RB. Multiplex PCR for the detection and quantification of zoonotic taxa of *Giardia*, *Cryptosporidium* and *Toxoplasma* in wastewater and mussels. Mol Cell Probes 2015;29:122–5. https://doi.org/10.1016/j.mcp.2015.01.001.
[135] Parsons MB, Travis D, Lonsdorf E V., Lipende I, Roellig DMA, Kamenya S, et al. Epidemiology and Molecular Characterization of *Cryptosporidium* spp. in Humans, Wild Primates, and Domesticated Animals in the Greater Gombe Ecosystem, Tanzania. PLoS Negl Trop Dis 2015;9:1–13. https://doi.org/10.1371/journal.pntd.0003529.
[136] Pitkänen T, Juselius T, Isomäki E, Miettinen IT, Valve M, Kivimäki AL, et al. Drinking water quality and occurrence of *Giardia* in finnish small groundwater supplies. Resources 2015;4:637–54. https://doi.org/10.3390/resources4030637.
[137] Prystajecky NA, Tsui CKM, Hsiao WWL, Uyaguari-Diaz MI, Ho J, Tang P, et al. *Giardia* spp. are commonly found in mixed assemblages in surface water, as revealed by molecular and whole-genome characterization. Appl Environ Microbiol 2015;81:4827–34. https://doi.org/10.1128/AEM.00524-15.
[138] Shanan S, Abd H, Bayoumi M, Saeed A, Sandström G. Prevalence of protozoa species in drinking and environmental water sources in Sudan. Biomed Res Int 2015;2015. https://doi.org/10.1155/2015/345619.
[139] Spanakos G, Biba A, Mavridou A, Karanis P. Occurrence of *Cryptosporidium* and *Giardia* in recycled waters used for irrigation and first description of *Cryptosporidium parvum* and *C. muris* in Greece. Parasitol Res 2015;114:1803–10. https://doi.org/10.1007/s00436-015-4366-6.
[140] Wells B, Shaw H, Hotchkiss E, Gilray J, Ayton R, Green J, et al. Prevalence, species identification and genotyping *Cryptosporidium* from livestock and deer in a catchment in the Cairngorms with a history of a contaminated public water supply. Parasites and Vectors 2015;8:1–13. https://doi.org/10.1186/s13071-015-0684-x.
[141] Koloren Z, Seferoğlu O, Karanis P. Occurency of *Giardia duodenalis* assemblages in river water sources of Black Sea, Turkey. Acta Trop 2016;164:337–44. https://doi.org/10.1016/j.actatropica.2016.09.025.
[142] Kumar T, Majid MAA, Onichandran S, Jaturas N, Andiappan H, Salibay CC, et al. Presence of *Cryptosporidium parvum* and *Giardia lamblia* in water samples from Southeast Asia: Towards an integrated water detection system. Infect Dis Poverty 2016;5:1–12. https://doi.org/10.1186/s40249-016-0095-z.
[143] Lapen DR, Schmidt PJ, Thomas JL, Edge TA, Flemming C, Keithlin J, et al. Towards a more accurate quantitative assessment of seasonal *Cryptosporidium* infection risks in surface waters using species and genotype information. Water Res 2016;105:625–37. https://doi.org/10.1016/j.watres.2016.08.023.
[144] Santos SFO, Silva HD, Wosnjuk LAC, Anunciação CE, Silveira-Lacerda EP, Peralta RHS, et al. Occurrence and Evaluation of

Methodologies to Detect *Cryptosporidium* spp. in Treated Water in the Central-West Region of Brazil. Expo Heal 2016;8:117–23. https://doi.org/10.1007/s12403-015-0187-1.
[145] Triviño-Valencia J, Lora F, Zuluaga JD, Gomez-Marin JE. Detection by PCR of pathogenic protozoa in raw and drinkable water samples in Colombia. Parasitol Res 2016;115:1789–97. https://doi.org/10.1007/s00436-016-4917-5.
[146] Ulloa-Stanojlović FM, Aguiar B, Jara LM, Sato MIZ, Guerrero JA, Hachich E, et al. Occurrence of *Giardia intestinalis* and *Cryptosporidium* sp. in wastewater samples from São Paulo State, Brazil, and Lima, Peru. Environ Sci Pollut Res 2016;23:22197–205. https://doi.org/10.1007/s11356-016-7537-9.
[147] Berglund B, Dienus O, Sokolova E, Berglind E, Matussek A, Pettersson T, et al. Occurrence and removal efficiency of parasitic protozoa in Swedish wastewater treatment plants. Sci Total Environ 2017;598:821–7. https://doi.org/10.1016/j.scitotenv.2017.04.015.
[148] Hatam-Nahavandi K, Mohebali M, Mahvi AH, Keshavarz H, Mirjalali H, Rezaei S, et al. Subtype analysis of *Giardia duodenalis* isolates from municipal and domestic raw wastewaters in Iran. Environ Sci Pollut Res 2017;24:12740–7. https://doi.org/10.1007/s11356-016-6316-y.
[149] Imre K, Sala C, Morar A, Ilie MS, Plutzer J, Imre M, et al. *Giardia duodenalis* and *Cryptosporidium* spp. as contaminant protozoa of the main rivers of western Romania: genetic characterization and public health potential of the isolates. Environ Sci Pollut Res 2017;24:18672–9. https://doi.org/10.1007/s11356-017-9543-y.
[150] Imre K, Morar A, Ilie MS, Plutzer J, Imre M, Emil T, et al. Survey of the Occurrence and Human Infective Potential of *Giardia duodenalis* and *Cryptosporidium* spp. in Wastewater and Different Surface Water Sources of Western Romania. Vector-Borne Zoonotic Dis 2017;17:685–91. https://doi.org/10.1089/vbz.2017.2155.
[151] Lass A, Szostakowska B, Korzeniewski K, Karanis P. Detection of *Giardia intestinalis* in water samples collected from natural water reservoirs and wells in northern and north-eastern Poland using LAMP, real-time PCR and nested PCR. J Water Health 2017;15:775–87. https://doi.org/10.2166/wh.2017.039.
[152] Ramo A, Del Cacho E, Sánchez-Acedo C, Quílez J. Occurrence and genetic diversity of *Cryptosporidium* and *Giardia* in urban wastewater treatment plants in north-eastern Spain. Sci Total Environ 2017;598:628–38. https://doi.org/10.1016/j.scitotenv.2017.04.097.
[153] Ramo A, Del Cacho E, Sánchez-Acedo C, Quílez J. Occurrence of *Cryptosporidium* and *Giardia* in raw and finished drinking water in north-eastern Spain. Sci Total Environ 2017;580:1007–13. https://doi.org/10.1016/j.scitotenv.2016.12.055.
[154] Xiao S, Yin P, Zhang Y, Hu S. Occurrence of *Cryptosporidium* and *Giardia* and the relationship between protozoa and water quality indicators in swimming pools. Korean J Parasitol 2017;55:129–36. https://doi.org/10.3347/kjp.2017.55.2.129.
[155] Bautista M, Bonatti TR, Fiuza VR d. S, Terashima A, Canales-Ramos M, José J, et al. Occurrence and molecular characterization of *Giardia duodenalis* cysts and *Cryptosporidium* oocysts in raw water samples from the Rímac River, Peru. Environ Sci Pollut Res 2018;25:11454–67. https://doi.org/10.1007/s11356-018-1423-6.
[156] Daley K, Truelstrup Hansen L, Jamieson RC, Hayward JL, Piorkowski GS, Krkosek W, et al. Chemical and microbial characteristics of municipal drinking water supply systems in the Canadian Arctic. Environ Sci Pollut Res 2018;25:32926–37.

https://doi.org/10.1007/s11356-017-9423-5.
[157] de Araújo RS, Aguiar B, Dropa M, Razzolini MTP, Sato MIZ, de Souza Lauretto M, et al. Detection and molecular characterization of *Cryptosporidium* species and *Giardia* assemblages in two watersheds in the metropolitan region of São Paulo, Brazil. Environ Sci Pollut Res 2018;25:15191–203. https://doi.org/10.1007/s11356-018-1620-3.
[158] Kalinová J, Valenčáková A, Hatalová E, Danišová O, Trungelová M, Hromada R. Occurrence of *Cryptosporidium* in the water basins of nitra region, Slovakia. Acta Trop 2018;179:36–8. https://doi.org/10.1016/j.actatropica.2017.12.019.
[159] Ladeia WA, Martins FDC, Silva CFRE, Freire RL. Molecular surveillance of *Cryptosporidium* and *Giardia duodenalis* in sludge and spent filter backwash water of a water treatment plant. J Water Health 2018;16:857–60. https://doi.org/10.2166/wh.2018.040.
[160] Sánchez C, López MC, Galeano LA, Qvarnstrom Y, Houghton K, Ramírez JD. Molecular detection and genotyping of pathogenic protozoan parasites in raw and treated water samples from southwest Colombia. Parasites and Vectors 2018;11:1–11. https://doi.org/10.1186/s13071-018-3147-3.
[161] Xiao S, Yin P, Zhang Y, Zhao X, Sun L, Yuan H, et al. Occurrence, genotyping, and health risk of *Cryptosporidium* and *Giardia* in recreational lakes in Tianjin, China. Water Res 2018;141:46–56. https://doi.org/10.1016/j.watres.2018.05.016.
[162] Xiao S, Zhang Y, Zhao X, Sun L, Hu S. Presence and molecular characterization of *Cryptosporidium* and *Giardia* in recreational lake water in Tianjin, China: A preliminary study. Sci Rep 2018;8:1–8. https://doi.org/10.1038/s41598-018-20902-3.
[163] Zahedi A, Gofton AW, Greay T, Monis P, Oskam CL, Ball A, et al. Profiling the diversity of *Cryptosporidium* species and genotypes in wastewater treatment plants in Australia using next generation sequencing. Sci Total Environ 2018;644:635–48. https://doi.org/10.1016/j.scitotenv.2018.07.024.
[164] Andrade RC, Bastos RKX, Bevilacqua PD, Andrade R V. *Cryptosporidium* genotyping and land use mapping for hazard identification and source tracking in a small mixed rural–urban watershed in Southeastern Brazil. J Water Health 2019;17:149–59. https://doi.org/10.2166/wh.2018.143.
[165] Hamdy D, El-Badry A, El Wahab WA. Assessment of *Giardia* and *Cryptosporidium* assemblages/species and their viability in potable tap water in Beni-Suef, Egypt using nested PCR/RFLP and staining. Iran J Parasitol 2019;14:368–78. https://doi.org/10.18502/ijpa.v14i3.1475.
[166] Ma L, Zhang X, Jian Y, Li X, Wang G, Hu Y, et al. Correction to: detection of *Cryptosporidium* and *Giardia* in the slaughterhouse, sewage and river waters of the Qinghai Tibetan plateau area (QTPA), China. Parasitol Res 2019;118:3571. https://doi.org/10.1007/s00436-019-06518-0.
[167] Masina S, Shirley J, Allen J, Sargeant JM, Guy RA, Wallis PM, et al. Weather, environmental conditions, and waterborne *Giardia* and *Cryptosporidium* in lqaluit, Nunavut. J Water Health 2019;17:84–97. https://doi.org/10.2166/wh.2018.323.
[168] Pignata C, Bonetta S, Bonetta S, Cacciò SM, Sannella AR, Gilli G, et al. *Cryptosporidium* oocyst contamination in drinking water: A case study in Italy. Int J Environ Res Public Health 2019;16:1–10. https://doi.org/10.3390/ijerph16112055.
[169] Utaaker KS, Joshi H, Kumar A, Chaudhary S, Robertson LJ. Occurrence of *Cryptosporidium* and *Giardia* in potable water

sources in Chandigarh, Northern India. J Water Supply Res Technol - AQUA 2019;68:483–94. https://doi.org/10.2166/aqua.2019.157.
[170] Abd El-Latif NF, El-Taweel HA, Gaballah A, Salem AI, Abd El-Malek AHM. Molecular Characterization of *Giardia intestinalis* Detected in Humans and Water Samples in Egypt. Acta Parasitol 2020;65:482–9. https://doi.org/10.2478/s11686-020-00176-4.
[171] Alhayali NS, Al-Amery AM, Hasan MH. Detection of *Giardia intestinalis* in human , calves and water supplies by traditional and molecular methods at Baghdad city, Iraq. Iraqi J Agric Sci 2020;51:1428–35.
[172] Benito M, Menacho C, Chueca P, Ormad MP, Goñi P. Seeking the reuse of effluents and sludge from conventional wastewater treatment plants: Analysis of the presence of intestinal protozoa and nematode eggs. J Environ Manage 2020;261. https://doi.org/10.1016/j.jenvman.2020.110268.
[173] Brooks YM, Spirito CM, Bae JS, Hong A, Mosier EM, Sausele DJ, et al. Fecal indicator bacteria, fecal source tracking markers, and pathogens detected in two Hudson River tributaries. Water Res 2020;171:115342. https://doi.org/10.1016/j.watres.2019.115342.
[174] Jiang W, Roellig DM, Li N, Wang L, Guo Y, Feng YY, et al. Contribution of hospitals to the occurrence of enteric protists in urban wastewater. Parasitol Res 2020;119:3033–40. https://doi.org/10.1007/s00436-020-06834-w.
[175] Ligda P, Claerebout E, Casaert S, Robertson LJ, Sotiraki S. Investigations from Northern Greece on mussels cultivated in areas proximal to wastewaters discharges, as a potential source for human infection with *Giardia* and *Cryptosporidium*. Exp Parasitol 2020;210:107848. https://doi.org/10.1016/j.exppara.2020.107848.
[176] Ligda P, Claerebout E, Kostopoulou D, Zdragas A, Casaert S, Robertson LJ, et al. *Cryptosporidium* and *Giardia* in surface water and drinking water: Animal sources and towards the use of a machine-learning approach as a tool for predicting contamination. Environ Pollut 2020;264:114766. https://doi.org/10.1016/j.envpol.2020.114766.
[177] Rusiñol M, Martínez-Puchol S, Timoneda N, Fernández-Cassi X, Pérez-Cataluña A, Fernández-Bravo A, et al. Metagenomic analysis of viruses, bacteria and protozoa in irrigation water. Int J Hyg Environ Health 2020;224:1–11. https://doi.org/10.1016/j.ijheh.2019.113440.
[178] Stokdyk JP, Firnstahl AD, Walsh JF, Spencer SK, de Lambert JR, Anderson AC, et al. Viral, bacterial, and protozoan pathogens and fecal markers in wells supplying groundwater to public water systems in Minnesota, USA. Water Res 2020;178:115814. https://doi.org/10.1016/j.watres.2020.115814.
[179] Atnafu B, Desta A, Assefa F. Microbial Community Structure and Diversity in Drinking Water Supply, Distribution Systems as well as Household Point of Use Sites in Addis Ababa City, Ethiopia. Microb Ecol 2021:1–17. https://doi.org/10.1007/s00248-021-01819-3.
[180] Barnes AN, Davaasuren A, Baasandavga U, Lantos PM, Gonchigoo B, Gray GC. Zoonotic enteric parasites in Mongolian people, animals, and the environment: Using One Health to address shared pathogens. PLoS Negl Trop Dis 2021;15:1–24. https://doi.org/10.1371/journal.pntd.0009543.
[181] Bivins A, Lowry S, Wankhede S, Hajare R, Murphy HM, Borchardt M, et al. Microbial water quality improvement associated

with transitioning from intermittent to continuous water supply in Nagpur, India. Water Res 2021;201:1–11. https://doi.org/10.1016/j.watres.2021.117301.
[182] Cervero-Aragó S, Desvars-Larrive A, Lindner G, Sommer R, Häfeli I, Walochnik J. Surface waters and urban brown rats as potential sources of human-infective *Cryptosporidium* and *Giardia* in Vienna, Austria. Microorganisms 2021;9:1–14. https://doi.org/10.3390/microorganisms9081596.
[183] dela Peña LBRO, Vejano MRA, Rivera WL. Molecular surveillance of *Cryptosporidium* spp. for microbial source tracking of fecal contamination in Laguna Lake, Philippines. J Water Health 2021;19:534–44. https://doi.org/10.2166/wh.2021.059.
[184] Falohun OO, Ayinmode AB, Adejinmi JO. Molecular characterisation of *Cryptosporidium* isolates from rivers, water treatment plants and abattoirs in Ibadan, Nigeria. Comp Immunol Microbiol Infect Dis 2021;74:1–6. https://doi.org/10.1016/j.cimid.2020.101577.
[185] Fan Y, Wang X, Yang R, Zhao W, Li N, Guo Y, et al. Molecular characterization of the waterborne pathogens *Cryptosporidium* spp., *Giardia duodenalis*, Enterocytozoon bieneusi, Cyclospora cayetanensis and Eimeria spp. in wastewater and sewage in Guangzhou, China. Parasit Vectors 2021;14:66. https://doi.org/10.1186/s13071-020-04566-5.
[186] Fernández-Baca CP, Spirito CM, Bae JS, Szegletes ZM, Barott N, Sausele DJ, et al. Rapid qPCR-Based Water Quality Monitoring in New York State Recreational Waters. Front Water 2021;3:1–19. https://doi.org/10.3389/frwa.2021.711477.
[187] Keenum I, Medina MC, Garner E, Pieper KJ, Blair MF, Milligan E, et al. Source-to-Tap Assessment of Microbiological Water Quality in Small Rural Drinking Water Systems in Puerto Rico Six Months After Hurricane Maria. Environ Sci Technol 2021;55:3775–85. https://doi.org/10.1021/acs.est.0c08814.
[188] Liu P, Amin N, Miah R, Foster T, Raj S, Corpuz MJB, et al. A method for correcting underestimation of enteric pathogen genome quantities in environmental samples. J Microbiol Methods 2021;189:1–10. https://doi.org/10.1016/j.mimet.2021.106320.
[189] Mphephu MG, Ekwanzala MD, Momba MNB. *Cryptosporidium* species and subtypes in river water and riverbed sediment using next-generation sequencing. Int J Parasitol 2021;51:339–51. https://doi.org/10.1016/j.ijpara.2020.10.005.
[190] Reiling SJ, Merks H, Zhu S, Boone R, Corneau N, Dixon BR. A cloth-based hybridization array system for rapid detection of the food- and waterborne protozoan parasites *Giardia duodenalis*, *Cryptosporidium* spp. and *Toxoplasma gondii*. Food Waterborne Parasitol 2021;24:1–8. https://doi.org/10.1016/j.fawpar.2021.e00130.
[191] Sun A, Stanton J-AL, Bergquist PL, Sunna A. Universal Enzyme-Based Field Workflow for Rapid and Sensitive Quantification of Water Pathogens. Microorganisms 2021;9:2367. https://doi.org/10.3390/microorganisms9112367.
[192] Vejano MRA, dela Peña LBRO, Rivera WL. Occurrence of *Giardia duodenalis* in selected stations and tributary rivers of Laguna Lake, Philippines. Environ Monit Assess 2021;193:466. https://doi.org/10.1007/s10661-021-09240-6.
[193] Yanta CA, Bessonov K, Robinson G, Troell K, Guy RA. CryptoGenotyper: A new bioinformatics tool for rapid *Cryptosporidium* identification. Food Waterborne Parasitol 2021;23:1–14. https://doi.org/10.1016/j.fawpar.2021.e00115.
